# Controllable Carbon Shell Encapsulation via Rapid Joule Heating Calcination for High‐Performance Asymmetric Supercapacitor With Suppressed Self‐Discharge and Robust Cycling Stability

**DOI:** 10.1002/advs.76184

**Published:** 2026-06-18

**Authors:** Qiang Zhou, Zheng Yang, Zhen Cao, Xulan Zheng, Xiao Yan, Yunsong Li, Xinsheng Zhao, Yuxiao Lin, Xiaoxiao Li

**Affiliations:** ^1^ School of Physics and Electronic Engineering Jiangsu Normal University Xuzhou China; ^2^ School of Chemistry and Materials Science Jiangsu Normal University Xuzhou China; ^3^ Zhejiang Laboratory Hangzhou China

**Keywords:** asymmetric supercapacitors, carbon shell, cycling stability, Joule heating, self‐discharge

## Abstract

Asymmetric supercapacitors (ASCs) comprising two different pseudocapacitive electrodes offer a promising route toward higher energy density, yet the serious self‐discharge behavior and poor cycle life hinder their wider applications. This work proposes a controllable carbon shell encapsulation strategy based on rapid Joule heating calcination to construct high‐performance ASC with suppressed self‐discharge and robust cycling stability. As a result, the as‐assembled ASC (H‐Fe_3_O_4_@C‐15//H‐NiCo_2_S_4_@C‐40) exhibits a maximum energy density of 105.6 W h kg^−1^ at a power density of 749 W kg^−1^, and long cycling lifespan with 93.7% capacitance retention after 25 000 cycles. Furthermore, current ASC also demonstrates moderated self‐discharge, with its open‐circuit voltage decaying from 1.48 to 0.75 V over 31102 s. Theoretically, the density functional theory (DFT) results adequately uncover that the significantly improved self‐discharge performance should originate from the increased adsorption energy between the electrode and the electrolyte ions. Meaningfully, this controllable carbon shell encapsulation strategy represents a universal and feasible approach to effectively suppress self‐discharge and extend the cycle life of ASC.

## Graphical abstract

The H‐Fe_3_O_4_@C negative electrode and H‐NiCo_2_S_4_@C positive electrode are designed through a controllable carbon shell encapsulation strategy. The as‐constructed asymmetric supercapacitor presents robust cycling stability with 93.7% capacity retention after 25 000 cycles. Furthermore, benefiting from increased adsorption energy between electrode and electrolyte ions, the moderating self‐discharge behavior is discovered (open‐circuit voltage drops from 1.48 to 0.75 V over 31102 s).

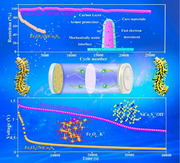

## 1 Introduction

At the forefront of research on electrochemical energy storage systems, the prime performance metrics for these technologies are reliability, energy and power density, cycle life. As a competitive energy storage device, supercapacitor (SC) effectively bridges the gap between conventional electrolytic capacitor and battery in terms of the above‐mentioned pivotal parameters, thereby playing an indispensable role in many fields such as energy recovery and grid regulation [1, 2]. Nevertheless, the relatively low energy density of SCs remains a major limitation to their broader application [3, 4]. According to the equation *E* = 1/2 *CV* ^2^, two strategies have been proposed to increase the energy density (*E*): i) developing novel electrode active materials for boosting device capacitance (*C*); ii) broadening the cell voltage (*V*) by employing different types of electrolytes or device configurations [5, 6, 7, 8, 9]. On the basis of this expression, a properly designed asymmetric supercapacitor (ASC), constructed from two distinct high‐capacity electrodes, can take full advantage of the different potential windows of each electrode to maximized the operating voltage of the full device, thus offering a promising route toward higher energy density [10, 11, 12].

In contrast to the electrostatic mechanism of electric double‐layer capacitors (EDLCs), pseudocapacitive electrode materials employ Faradaic processes for charge storage, which involve rapid and reversible redox reactions at or near the active material surface [13, 14]. Accordingly, the theoretical charge storage capacity of pseudocapacitors can be 10 to 100 times greater than that of EDLCs [8, 15, 16]. Recently, considerable research efforts have increasingly focused on the construction of ASCs by integrating two dissimilar pseudocapacitive electrodes that operate within distinct and complementary potential windows [15, 16, 17, 18, 19]. For instance, Peng et al. designed an ASC comprising MnO_2_/carbon nanotubes and MoS_2_/carbon nanotubes composites as positive and negative electrodes, exhibiting an extended working voltage of 1.8 V [20]. Similarly, benefiting from the well‐matched potential windows of SiC@Fe_2_O_3_ negative electrode and SiC@NiCo_2_O_4_/Ni(OH)_2_ positive electrode, the operating voltage of the designed ASC could be extended to 1.75 V, leading to a high energy density of 103 W h kg^−1^ for the device [15]. However, the thorny problem that has to be faced is that although pseudocapacitive electrode materials own high theoretical specific capacitance, their sluggish kinetics and intricate redox reactions lead to inferior rate capability, limited cycling stability, and rapid self‐discharge [21, 22, 23, 24, 25]. Ideally, to improve the electrochemical performance of ASCs constructed with different pseudocapacitive materials, a promising strategy is to encapsulate them in a core‐shell structure with a conductive carbon shell, which protects fragile pseudocapacitive materials and enhances their overall functionality [26, 27, 28].

Recently, the construction of core‐shell composites via carbon shell encapsulation has emerged as a prominent approach in designing high‐performance nanostructure materials for electrochemical energy storage and conversion applications [29, 30, 31, 32]. The introduction of an exterior carbon shell exerts both physical and chemical influences on the core pseudocapacitive material, leading to significantly enhanced functionality in the resulting core‐shell structure. Generally, a carbon shell is beneficial for achieving rapid electron transport and optimizing rate capability [33, 34, 35, 36, 37]. Moreover, the surface carbon layer spatially confines the volume change of the pseudocapacitive core, playing a critical role in mitigating pulverization of the active material [38]. Furthermore, unlike the complex surface states of pseudocapacitive materials, the carbon shell possesses a simpler and more stable surface, which can weaken the self‐discharge behavior caused by the loose‐bonding model and spontaneous Faraday reactions under open‐circuit condition [39]. It is well established that the thickness and graphitization degree of the surface carbon layer directly govern the physicochemical properties of core‐shell composites. An optimal combination of these parameter is essential for efficient mass transport and electron transfer. Typically, carbon shell generated at lower annealing temperatures exhibits limited graphitization. While elevating the temperature enhances crystallinity, excessive heat can trigger displacement reactions between the carbon and the pseudocapacitive core (e.g., metal oxide/sulfide/nitride), leading to structural degradation. Currently, a significant challenge lies in achieving controllable construction of coating thickness while increasing the graphitization degree of the carbon layer.

Fe_3_O_4_ and NiCo_2_S_4_ are representative pseudocapacitive materials with complementary operating potential windows (−1 to 0 V and 0 to 0.5 V, respectively) [12, 16]. As such, ASCs constructed from these materials or their derivatives have received considerable attention and demonstrated excellent high‐capacity characteristics [15]. However, achieving satisfactory cycling stability remains a significant challenge, and the self‐discharge behavior of these devices has rarely been explored. In this work, we design an advanced ASC using carbon shell coated Fe_3_O_4_ nanorods (H‐Fe_3_O_4_@C) and NiCo_2_S_4_ nanowires (H‐NiCo_2_S_4_@C) grown on carbon fabric (CF) substrate as negative and positive electrodes, respectively. The thickness and graphitization of surface carbon layer are optimized by regulating the electrodeposition of polymer precursors and the rapid Joule heating calcination. The as‐assembled aqueous‐based ASC presents high energy density (105.6 W h kg^−1^ with a power density of 749 W kg^−1^), robust cycling stability (93.7% capacity retention after 25 000 cycles), and suppressed self‐discharge (open‐circuit voltage drops from 1.48 to 0.75 V in 31102 s). Furthermore, the density functional theory (DFT) calculations indicate that the increased adsorption energy between the electrode and electrolyte ions contributes significantly to the suppression of self‐discharge.

## 2 Results and Discussion

**SCHEME 1:**
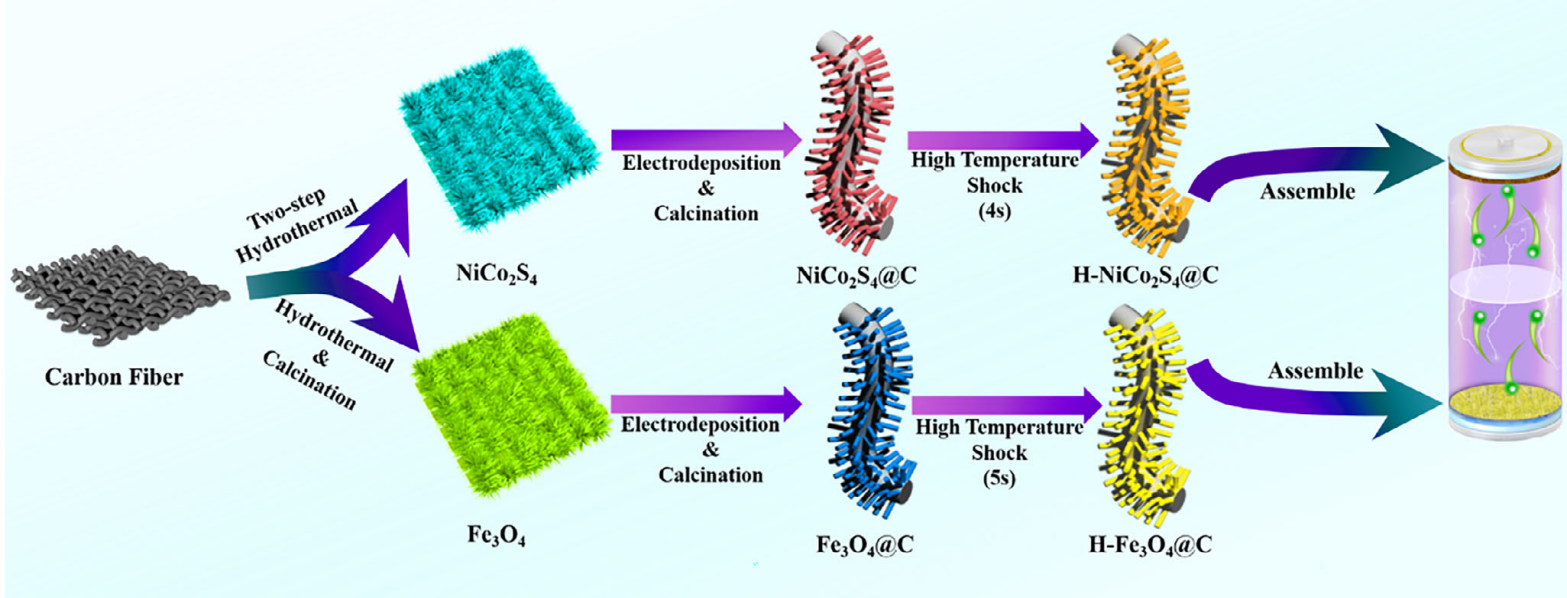
Schematic illustration on the preparation procedures of H‐NiCo_2_S_4_@C positive electrode and H‐Fe_3_O_4_@C negative electrode grown on the CF.

The representative preparation procedures of H‐Fe_3_O_4_@C negative electrode and H‐NiCo_2_S_4_@C positive electrode are schematically displayed in Scheme 1 (see the details in Experimental Section, Supporting Information). First, the primordial Fe_3_O_4_ nanorods and NiCo_2_S_4_ nanowires were grown in situ on CF substrate via hydrothermal‐calcination and two‐step hydrothermal reactions, respectively. Then, the target H‐Fe_3_O_4_@C and H‐NiCo_2_S_4_@C electrodes with optimized surface carbon layer thickness and graphitization were fabricated by sequentially subjecting the pristine nanorods and nanowires to electrodeposition of polymer precursor, low‐temperature annealing, and rapid Joule heating calcination.

**FIGURE 1:**
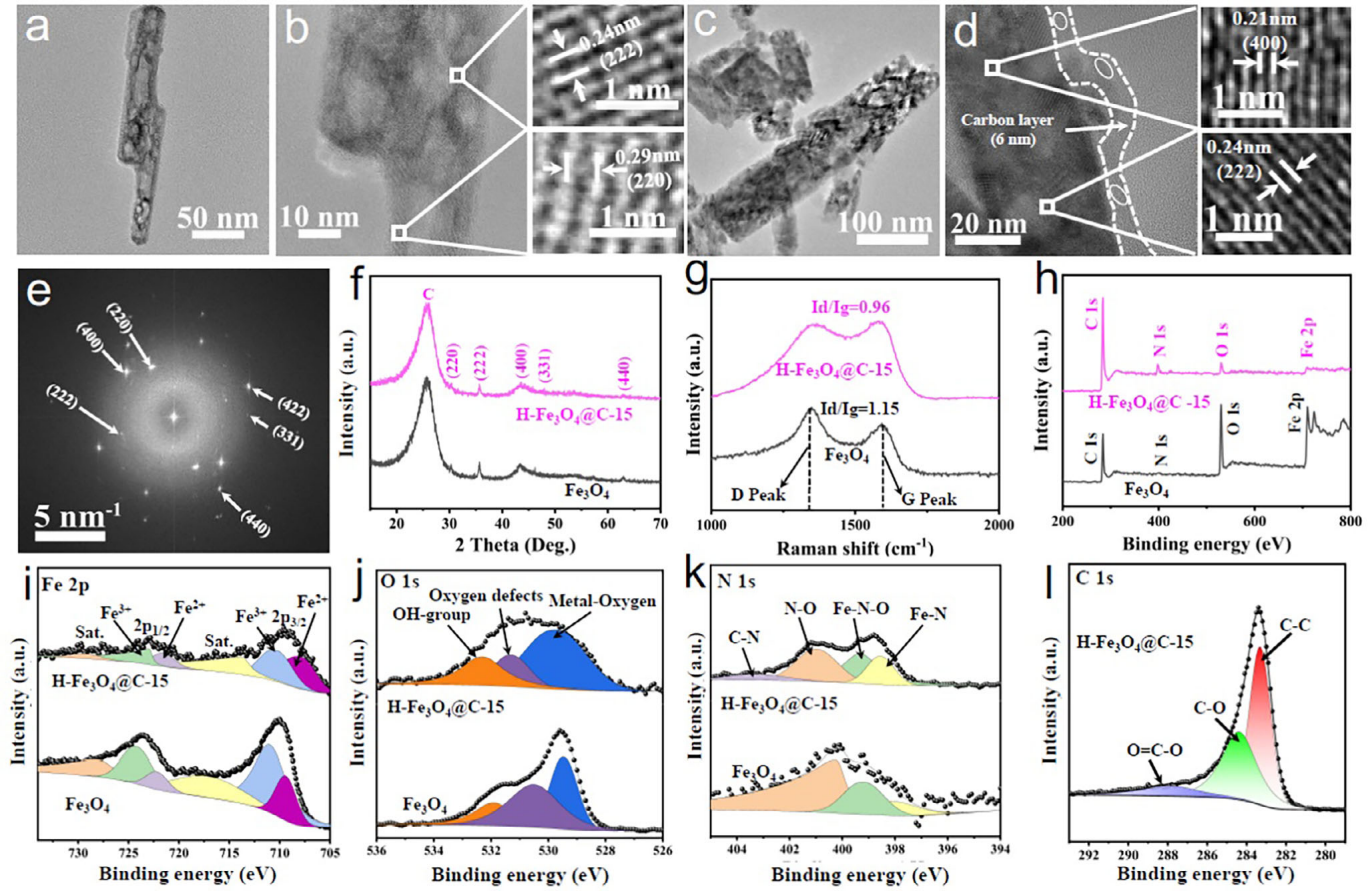
Typical TEM and HRTEM images of Fe_3_O_4_ (a and b) and H‐Fe_3_O_4_@C‐15 (c and d). (e) SAED pattern of the H‐Fe_3_O_4_@C‐15. (f, g) XRD and Raman patterns of Fe_3_O_4_ and H‐Fe_3_O_4_@C‐15 on the CF substrate. XPS survey spectrum (h) and high‐resolution XPS spectra of Fe 2p, O 1s, N 1s, C 1s (i‐l) for Fe_3_O_4_ and H‐Fe_3_O_4_@C‐15.

Figure S1 shows the scanning electron microscope (SEM) images of Fe_3_O_4_ (Figure S1a) and H‐Fe_3_O_4_@C‐t (t = 10, 15, 20, referred to the time of electrodeposition reaction, Figure S1b–d) under different magnifications, respectively. The results display that the fine nanorods are uniformly covered on the surface of CF substrate. Notably, the subsequent electrodeposition and sintering processes do not alter the original morphology and uniformity of the nanostructure. The transmission electron microscope (TEM) image of Fe_3_O_4_ products (Figure 1a) clearly reveals their rod‐like structure with typical length of ∼150 nm and diameter of ∼40 nm. The corresponding high‐resolution TEM (HRTEM) images (Figure 1b) disclose the adjacent lattice spacings of 0.24 and 0.29 nm, which correspond to the (222) and (220) crystal planes of Fe_3_O_4_, clarifying the polycrystalline nature of products [40]. Figure 1c gives the TEM image of resultant sample with 15 min electrodeposition of polymer precursors followed by low‐temperature annealing and rapid Joule heating calcination (i.e., H‐Fe_3_O_4_@C‐15), implying the consistency of nanostructure. Further observation, a carbon layer with a thickness of ∼6 nm is visible in the HRTEM image (Figure 1d), intimately attached to the surface of Fe_3_O_4_ nanorods. In detail, the carbon shell features numerous layered nanostructured, resembling those in hard carbon. This short‐range ordered architecture facilitates rapid ion transport and good electrical conductivity, thereby endowing the electrode with superior rate capability and high Coulombic efficiency [41]. Simultaneously, it enhances mechanical protection of the core, leading to improved cycling stability [42]. Besides, the (400) and (222) crystal planes of Fe_3_O_4_ with interplanar spacings of 0.21 and 0.24 nm are discovered, indicating that the preparation of the carbon layer does not affect the prior grown counterparts. For comparison, the TEM and HRTEM images of H‐Fe_3_O_4_@C‐10 (Figure S2a,b) and H‐Fe_3_O_4_@C‐20 (Figure S2c,d) are shown, and a morphology similar to H‐Fe_3_O_4_@C‐15 is displayed. Whereas, the thickness of the coated carbon layer shows an increasing trend with the extension of electrodeposition time. In addition, the clear diffraction rings once again reveal the polycrystalline characteristics of the Fe_3_O_4_ core (Figure 1e). The elemental mapping images (Figure S3) show a relatively homogeneous distribution of Fe, N, O, and C elements in H‐Fe_3_O_4_@C‐15.

Figure 1f,g shows the x‐ray diffraction (XRD) patterns and Raman spectra of the Fe_3_O_4_ and H‐Fe_3_O_4_@C‐15 samples, respectively. Except for the high‐intensity peak of the CF substrates at 2θ = 26°, all the other diffraction peaks are well indexed to Fe_3_O_4_ (PDF#86‐1340), indicating that the phase composition does not change after the rapid Joule heating calcination. According to the Raman spectra (Figure 1g), H‐Fe_3_O_4_@C‐15 exhibits an I_D_/I_G_ ratio of 0.96, indicating the carbon shell composed of disordered carbon incorporating numerous localized graphitic zones, which is consistent with the observation results of HRTEM (Figure 1d) [43, 44]. Moreover, the I_D_/I_G_ ratio of 1.15 detected in Fe_3_O_4_ sample should be attributed to the CF substrate. The x‐ray photoelectron spectroscopy (XPS) studies the differences in the surface chemical composition between Fe_3_O_4_ and H‐Fe_3_O_4_@C‐15 (Figure 1h–l). The C (285 eV), N (398 eV), Fe (709 eV), and O (531 eV) elements are detected within two electrodes (Figure 1h) (The C signal of pure Fe_3_O_4_ originates from CF substrate). The corresponding atomic concentrations of the Fe_3_O_4_ and H‐Fe_3_O_4_@C‐15 are summarized in Table S3. The atomic ratio of Fe to O is close to 3:4, confirming that the core material is Fe_3_O_4_. The increased content of C further verifies the successful coating of the carbon layer. In the high‐resolution Fe 2p spectra of two samples (Figure 1i), two main peaks Fe 2p_3/2_ and Fe 2p_1/2_ center at 710.3 and 724.0 eV, as well as two corresponding satellites at 718.5 and 732.5 eV (marked as sat.) can be detected. Precisely, the Fe 2p_3/2_ and Fe 2p_1/2_ can be fitted into Fe^2+^ and Fe^3+^ [19, 45]. Furthermore, the higher Fe^2+^/Fe^3+^ ratio in H‐Fe_3_O_4_@C‐15 compared to pristine Fe_3_O_4_ indicates that a portion of Fe^3+^ was reduced to Fe^2+^ through a reduction with external carbon layer during the thermal process [46]. The high‐resolution O 1s spectra of two samples (Figure 1j) shows three binding energies at 532.3 eV (OH‐group, surface physical or chemical adsorption of water), 531.5 eV (oxygen defect) and 529.7 eV (metal oxygen) [17, 44]. Compared with the oxygen distribution of Fe_3_O_4_, H‐Fe_3_O_4_@C‐15 exhibits a higher proportion of OH‐group and metal Fe─O bonds but a lower concentration of oxygen defects. The increased surface OH‐group maybe beneficial to improve electrode wettability [47]. Moreover, the rise in metal‐oxygen bonds and concurrent decrease in oxygen defects suggest the structural reorganization toward higher crystallinity, accompanied by a lower concentration of surface functional groups [48]. The N 1s spectrum of Fe_3_O_4_ (Figure 1k) is deconvoluted into three peaks of 398.5 eV (Fe─N), 399.3 eV (Fe─N─O), 400.8 eV (N─O), revealing the N dopants are incorporated into the crystal lattices of Fe_3_O_4_ [49]. The extra C─N (403.3 eV) in H‐Fe_3_O_4_@C‐15 suggests that some N atoms have entered the lattice of the surface carbon layer, which is conducive to improving the electrical conductivity of electrode [49, 50, 51]. The C 1s spectrum of H‐Fe_3_O_4_@C‐15 (Figure 1l) can be divided into strong C─C bond (283.5 eV) as well as weak C─O (284.5 eV) and O═C─O (288 eV) signal peaks, indicating that the surface of H‐Fe_3_O_4_@C‐15 samples is clean with fewer carbon‐based functional groups [31].

**FIGURE 2:**
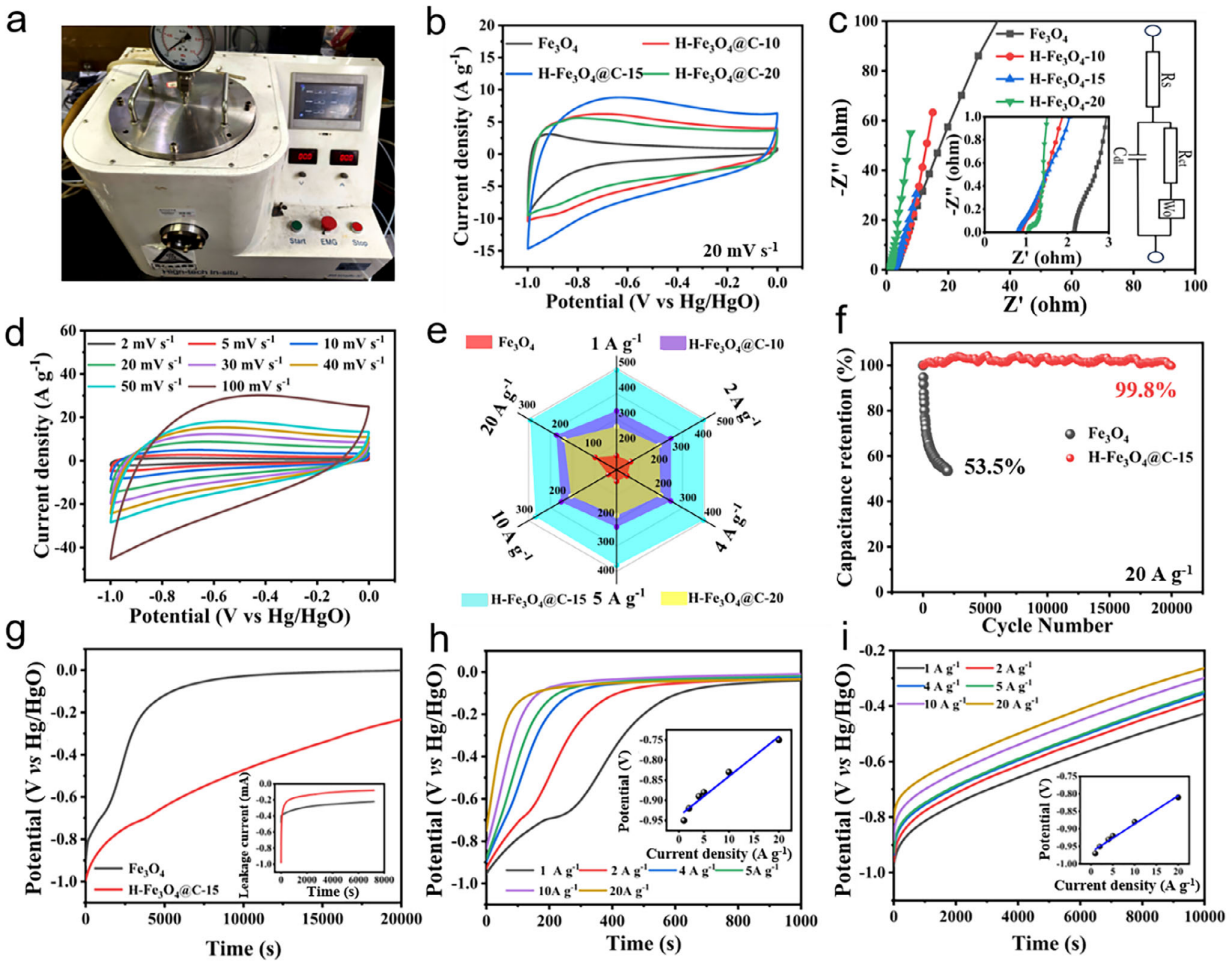
(a) Digital photograph of the rapid Joule heating instrument. (b, c) The CV and Nyquist curves of Fe_3_O_4_ and H‐Fe_3_O_4_@C‐t electrodes, the insets show the details in high‐frequency range and electrical equivalent circuit. (d) The CV curves of H‐Fe_3_O_4_@C‐15 at different scan rates. (e) The specific capacitances in radar map of various electrodes versus current densities. (f) The cycling stability of the Fe_3_O_4_ and H‐Fe_3_O_4_@C‐15 electrodes tested using GCD at a current density of 20 A g^−1^. Self‐discharge performance of Fe_3_O_4_ and H‐Fe_3_O_4_@C‐15 electrodes: (g) decay of open‐circuit potential after holding potential at −1.0 V for 2 h and the inset is corresponding to the various leakage currents.; decay of open‐circuit potential for the (h) Fe_3_O_4_ and (i) H‐Fe_3_O_4_@C‐15 electrodes after being charged with different current densities from 1 to 20 A g^−1,^ the insets show the relationship between the initial voltage and charging current density.

Figure 2a is the digital photograph of the rapid Joule heating instrument, and the temperature and time are controlled by adjusting the power supply current. The rapid Joule heating calcination simultaneously promotes carbon graphitization and prevents core‐shell interfacial reactions, which is key to the controllable preparation of high‐quality carbon layer. To evaluate the performance of Fe_3_O_4_‐based negative electrodes, the electrochemical measurements are performed under a standard three‐electrode configuration. The cyclic voltammetry (CV) and galvanostatic charging/discharging (GCD) curves of CF, Fe_3_O_4_, Fe_3_O_4_@C‐15 and H‐Fe_3_O_4_@C‐15 electrodes at 20 mV s^−1^ and 1 A g^−1^ (Figure S4) show that the capacitance contribution of CF is nearly negligible, while the H‐Fe_3_O_4_@C‐15 electrode exhibits a remarkable capacity enhancement. Figure 2b and Figure S5 present the comparison of CV and GCD curves of the Fe_3_O_4_ and H‐Fe_3_O_4_@C‐t at the same scan rate of 20 mV s^−1^ and a constant current density of 1 A g^−1^. Obviously, H‐Fe_3_O_4_@C‐15 exhibits the maximum integrated CV area and longest discharge time, suggesting its superior capacitance to all other samples [52]. Moreover, the CV curve shapes demonstrate that the charge storage originates from the EDLC induced by electrolyte ion surface adsorption, as well as the pseudocapacitance derived from the Fe^2+^/Fe^3+^ redox couples of Fe_3_O_4_ [16]. The Nyquist plots (Figure 2c) reveal that all internal resistances (the *x*‐intercepts of the curves) of H‐Fe_3_O_4_@C‐t electrodes are obviously lower than that of pure Fe_3_O_4_ electrode (2.16 Ω). The smallest internal resistance of 0.82 Ω is detected at the given deposition time of 15 min, and then it slightly rises to 1.04 Ω at the further prolonged deposition time of 20 min, which maybe attributable to the overly thick carbon layer hinders the transmission of electrons [53]. As summarized in Table S4, the H‐Fe_3_O_4_@C‐15 electrode also exhibits the lowest interfacial resistance (the diameter of the semicircle in the middle frequency region, which relates to the ion migration across the interface between the electrolyte and electrode) of 0.68 Ω, demonstrating that the optimal carbon coating effectively reduces charge transfer barriers and enhances reaction kinetics. Additionally, the steeper low‐frequency slope observed in H‐Fe_3_O_4_@C‐t electrodes suggests the faster ion diffusion, which benefited from the optimized interfacial reaction kinetics. Figure 2d and Figure S6 illustrate the CV and GCD curves of H‐Fe_3_O_4_@C‐15 electrode at various scan rates and current densities. Their well‐maintained CV and GCD curve shapes suggest outstanding rate performance and Coulombic efficiency of the electrode [54]. The specific capacitance plot (Figure 2e) derived from the GCD curves of H‐Fe_3_O_4_@C‐t (Figures S6–S9) shows that the largest specific capacitance of 493 F g^−1^ (Table S1 shows the mass loading of H‐Fe_3_O_4_@C‐t electrodes) can be achieved for H‐Fe_3_O_4_@C‐15 electrode at a current density of 1 A g^−1^, which is more than three times to that of pure Fe_3_O_4_ (156 F g^−1^). Increasing the current density 20‐fold up to 20 A g^−1^, 52% of initial capacitance is retained, exhibiting the excellent rate capability of H‐Fe_3_O_4_@C‐15 electrode. Moreover, the energy storage analysis reveals a hybrid charge storage mechanism in present electrode, featuring a collaborative contribution from both diffusion‐controlled and capacitive processes (see the details in Figure S10) [11, 29]. Furthermore, the proportion of capacitive contribution increases from 25% to 93% as the scan rate increases from 2 to 50 mV s^−^^1^, while the diffusion‐controlled contribution decreases accordingly (Figures S11–S13). Notably, the charge storage is mainly governed by diffusion‐controlled processes at low scan rate, whereas the capacitive contribution becomes dominant at high scan rate, which can be attributed to the number of OH^−^ ions reaching to the active material surface decreases with increasing scan rate [55, 56]. Remarkably, the H‐Fe_3_O_4_@C‐15 electrode demonstrates exceptional stability (Figure 2f), retaining 99.8% of initial capacitance after 20 000 charge/discharge cycles, far exceeding the pure Fe_3_O_4_ electrode (53.5% retention after 2000 cycles). As evidenced by the post‐cycling SEM images (Figure S14), the H‐Fe_3_O_4_‐15 retains a well‐defined morphology, in stark contrast to the sparse and degraded structure of Fe_3_O_4_ resulting from electrolyte‐induced corrosion over the course of cycling. The present H‐Fe_3_O_4_@C‐15 electrode exhibits competitively high charge storage capacity and exceptional cycling stability, which surpasses that of most previously reported Fe_3_O_4_‐based electrodes (Table S5).

Assessing the self‐discharge performance of individual electrodes in a three‐electrode system is a fundamental prerequisite for developing high‐performance supercapacitors with long‐term energy retention [57, 58]. Figure 2g–i detects the self‐discharge behavior of Fe_3_O_4_ and H‐Fe_3_O_4_@C‐15 electrodes by the open‐circuit potential and leakage current measurements. To eliminate the influence of uneven charge distribution, the open‐circuit potential decay is monitored after the electrode is charged to −1 V at the 1 A g^−1^ and subsequently held at that potential for 2 h (Figure 2g). The H‐Fe_3_O_4_@C‐15 electrode demonstrates a significantly slower self‐discharge, with its open‐circuit potential decaying from −1 to −0.5 V over 9055s. This duration is 4.5 times longer than that of Fe_3_O_4_ electrode (reaches the same potential window in only 2121s), indicating markedly inhibited self‐discharge behavior. In addition, the leakage currents of both electrodes (the inset of Figure 2g) show a transient surge at the beginning, then gradually subsided to a stable baseline over time. The steady‐state current of H‐Fe_3_O_4_@C‐15 is lower than that of Fe_3_O_4_ (−100 *µA vs* −240 *µA*), corresponding to the result of open‐circuit potential decline. Figure 2h,i presents the open‐circuit potential decay profiles after charging the electrodes to −1 V at different currents densities (1, 2, 4, 5, 10, 20 A g^−1^). At a lower charging current density, the ion transport kinetics allows for equilibrium adsorption, forming a stable electric double layer; conversely, a higher charging current density causes rapid and random ion accumulation near the electrode surface, forming a less stable electric double layer [23]. As expected, the potential decay rate and initial potential of both electrodes have been improved at a low charging current density. A linear increase in initial potential is observed with decreasing current density (the insets of Figure 2h,i). Notably, the H‐Fe_3_O_4_@C‐15 electrode exhibits lower self‐discharge and higher initial potential than Fe_3_O_4_ at the same charging current density, which may be attribute to the enhanced interfacial affinity between its clean carbon surface and K^+^ [59].

**FIGURE 3:**
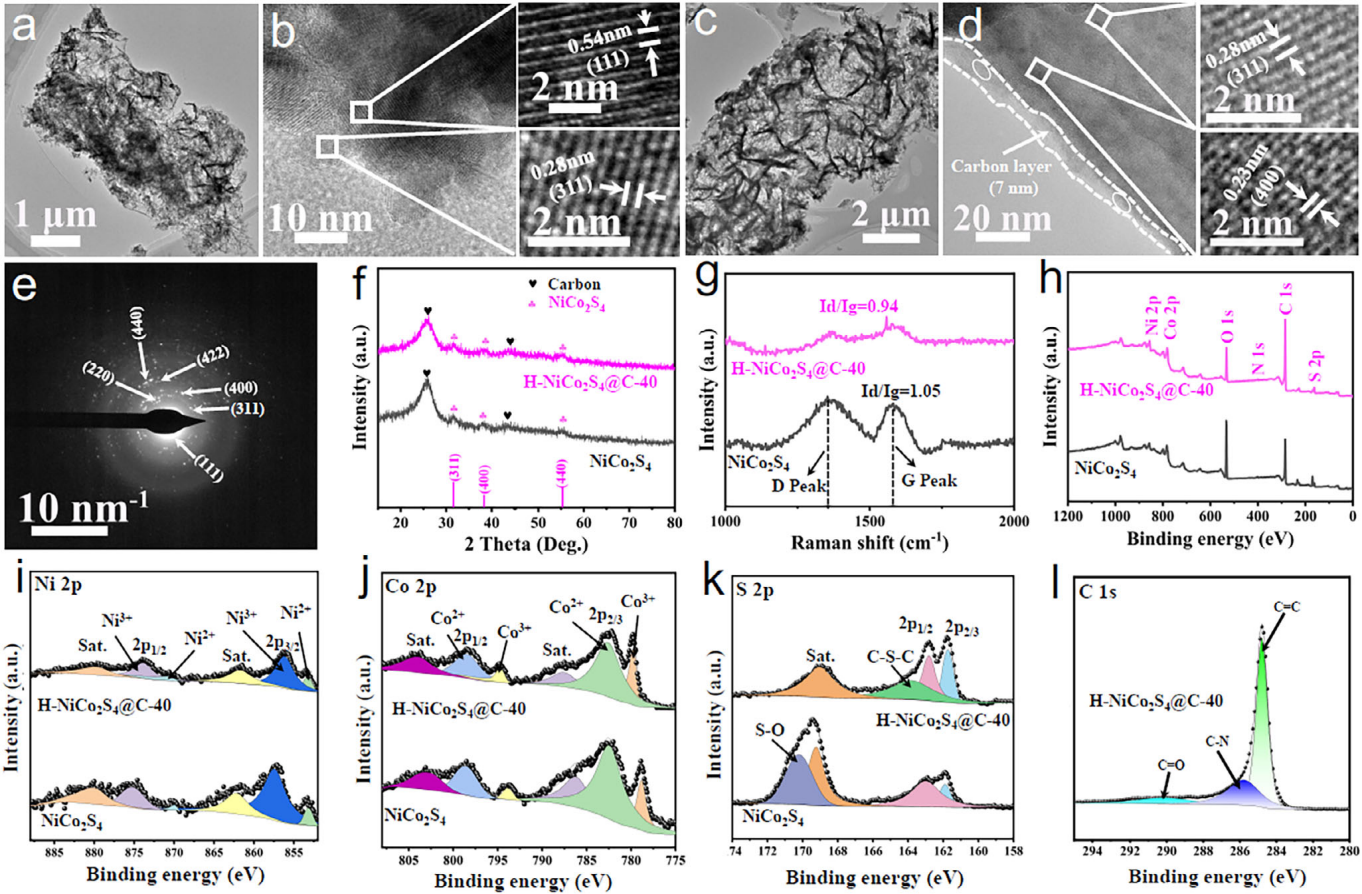
Typical TEM and HRTEM images of NiCo_2_S_4_ (a and b) and H‐NiCo_2_S_4_@C‐40 (c and d). (e) SAED pattern of the H‐NiCo_2_S_4_@C‐40. (f, g) XRD and Raman patterns of NiCo_2_S_4_ and H‐NiCo_2_S_4_@C‐40 on the CF substrate. XPS survey spectrum (h) and high‐resolution XPS spectra of Ni 2p, Co 2p, S 2p, C 1s (i–l) for NiCo_2_S_4_ and H‐NiCo_2_S_4_@C‐40.

For matching the working voltage of the H‐Fe_3_O_4_@C‐15 negative electrode, the H‐NiCo_2_S_4_@C electrode is prepared as the positive electrode to build a high‐performance ASC. The SEM images of NiCo_2_S_4_ (Figure S15a) and H‐NiCo_2_S_4_@C‐t (t = 20, 40, 60, referred to the time of electrodeposition reaction, Figure S15b–d) show that the CF substrate is uniformly covered with as‐synthesis nanowires, demonstrating their original morphology and uniformity remain unaffected by the subsequent electrodeposition and sintering processes. Figure 3a shows the TEM image of NiCo_2_S_4_ nanowires, and their polycrystallinity is evidenced by the corresponding HRTEM images (Figure 3b), which show well‐defined lattice spacings assignable to the (111) and (311) planes of NiCo_2_S_4_. Additionally, the TEM and HRTEM images of H‐NiCo_2_S_4_@C‐t (Figure 3c,d and Figure S16) confirm that the NiCo_2_S_4_ core is completely wrapped by a carbon shell, and the thickness of carbon layer increases with the extension of electrodeposition time. Analogous to the carbon shell on Fe_3_O_4_ nanorods mentioned above, the NiCo_2_S_4_ nanowire coating also contains abundant layered nanostructured regions. Moreover, the polycrystalline property of NiCo_2_S_4_ core is once again illustrated by the diffraction rings of H‐NiCo_2_S_4_@C‐40 (Figure 3e). The elemental mapping images (Figure S17) present uniform distribution for Ni, Co, S, C, N elements in H‐NiCo_2_S_4_@C‐40.

Figure 3f is the XRD patterns of NiCo_2_S_4_ and H‐NiCo_2_S_4_@C‐40 on CF substrate, indicating the as‐grown products are NiCo_2_S_4_, and the Joule heating calcination process does not affect the phase composition. The Raman spectra (Figure 3g) present an I_D_/I_G_ value of 1.05 in NiCo_2_S_4_, which comes from the CF substrate. A lower I_D_/I_G_ ratio in H‐NiCo_2_S_4_@C‐40 (0.94) is observed, reflecting the presence of short‐range ordered graphitic structure in the carbon shell. The XPS spectra in Figure 3h–l and Figure S18 illustrate the surface composition of Ni, Co, S, N, C, and O species and elemental chemical states within NiCo_2_S_4_ and H‐NiCo_2_S_4_@C‐40. Where, the O signals originate from partially oxidized and adsorbed water on the surface [60], and the C signal of pure NiCo_2_S_4_ originates from CF substrate. The atomic concentrations of samples are listed in Table S6. The atomic ratio of Ni: Co: S is approximately 1:2:4, indicating that the NiCo_2_S_4_ core material remains unchanged after the Joule heating treatment. Notably, the carbon content of H‐NiCo_2_S_4_‐40 is higher than that of pure NiCo_2_S_4_, which confirms the successful coating of the carbon layer. Comparing the Ni 2p and Co 2p spectra of two electrodes (Figure 3i,j), the ratio of Ni^2+^/Ni^3+^ and Co^2+^/Co^3+^ increased substantially, indicating that part of Ni^3+^ and Co^3+^ underwent a reduction reaction with external carbon shell to turn into Ni^2+^ and Co^2+^ [61, 62, 63, 64]. Furthermore, the S 2p spectrum of H‐NiCo_2_S_4_@C‐40 electrode exhibits a new C─S─C peak at 163.8 eV and the concomitant disappearance of the S─O peak (170.2 eV) observed in pristine NiCo_2_S_4_ electrode (Figure 3k). The added C─S─C peak signifies a stable core‐shell interface, facilitating efficient electron transfer between the carbon shell and NiCo_2_S_4_ core [65, 66]. Concurrently, the removal of S─O bonds reduces surface complexity with fewer functional groups, promoting direct ion adsorption onto the carbon layer and forming a tightly bound interface that mitigates ion desorption under open‐circuit conditions [58, 67, 68, 69]. The C 1s spectrum of H‐NiCo_2_S_4_@C‐40 (Figure 3l) indicates a simplified surface chemistry with three peaks of C═C (284.8 eV), C─N (285.7 eV) and C═O (290.2 eV) [40, 60]. The N 1s spectra of two electrodes (Figure S18) respond to more obvious signals of pyridinic‐N (398.7 eV), metal‐N (399.6 eV), pyrrolic‐N (400.5 eV) and graphitic‐N (401.5 eV) in H‐NiCo_2_S_4_@C‐40 than NiCo_2_S_4_. The detected metal‐N bonding indicates N doping into the NiCo_2_S_4_ [70, 71, 72]. Furthermore, the pyridinic‐N, pyrrolic‐N, and graphitic‐N configurations profoundly elevate the electron density of carbon shell and influence the interface of shell/core by improving ion transport kinetics [60, 73].

**FIGURE 4:**
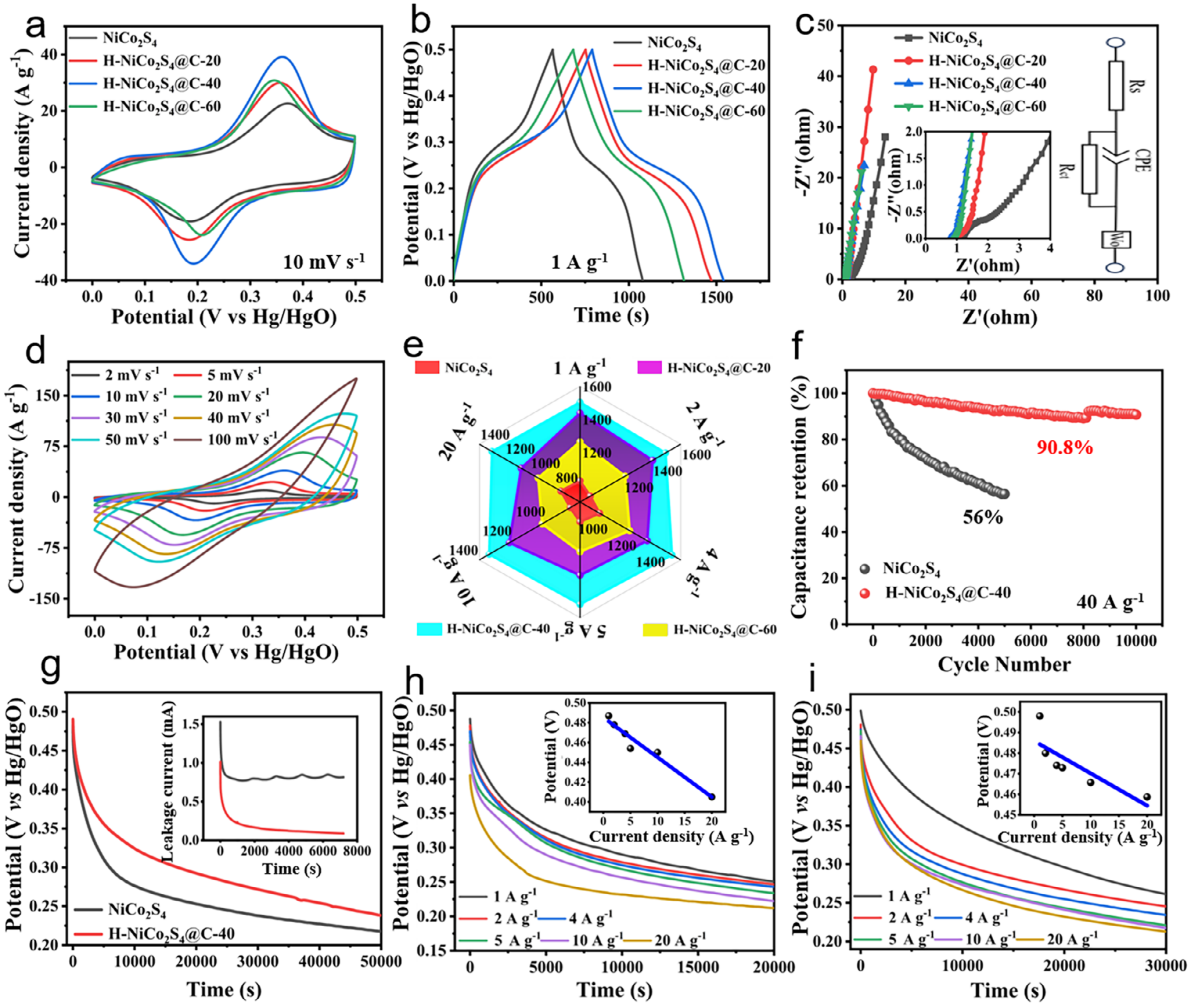
The electrochemical performance of NiCo_2_S_4_ and H‐NiCo_2_S_4_@C‐t electrodes: (a, b) CV and GCD curves at a scan rate of 10 mV s^−1^ and a current density of 1 A g^−1^; (c) Nyquist curves, the insets show the details in high‐frequency range and electrical equivalent circuit. (d) The CV curves of H‐NiCo_2_S_4_@C‐40 at different scan rates. (e) The specific capacitances in radar map of various electrodes versus current densities. (f) The cycling stability of the NiCo_2_S_4_ and H‐NiCo_2_S_4_@C‐40 electrodes tested using GCD at a current density of 40 A g^−1^. Self‐discharge performance of NiCo_2_S_4_ and H‐NiCo_2_S_4_@C‐40 electrodes: (g) decay of open‐circuit potential after holding potential at 0.5 V for 2 h and the inset is corresponding to the various leakage currents.; decay of open‐circuit potential for the (h) NiCo_2_S_4_ and (i) H‐NiCo_2_S_4_@C‐40 electrodes after being charged with different current densities from 1 to 20 A g^−1,^ the insets show the relationship between the initial voltage and charging current density.

The electrochemical behavior of NiCo_2_S_4_‐based positive electrodes is estimated under a three‐electrode system with 6.0 m KOH solution as electrolyte and Hg/HgO as the reference electrode. Figure S19 displays the CV and GCD curves of CF, NiCo_2_S_4,_ NiCo_2_S_4_@C‐40 and H‐NiCo_2_S_4_@C‐40 electrodes at a same scan rate of 20 mV s^−1^ and a constant current density of 1 A g^−1^, suggesting the rapid Joule heating roasting can further enhance the specific capacity of the composite electrode. The CV and GCD curves of NiCo_2_S_4_ and H‐NiCo_2_S_4_@C‐t at 10 mV s^−1^ and 1 A g^−1^ (Figure 4a,b) show that the H‐NiCo_2_S_4_@C‐40 electrode exhibits the maximum integrated CV area and longest discharge time, suggesting its superior capacitance to all other electrodes [74]. The well‐defined oxidation peaks appear at approximately 0.35 V, while the corresponding reduction peaks are located at around 0.20 V, reflecting the typical faradaic redox behavior of the electrode material, which are assigned to the reversible faradaic reactions originating from the Ni^2+^/Ni^3+^ and Co^2+^/Co^3+^ redox couples [7]. Their Nyquist plots (Figure 4c) present that all the H‐NiCo_2_S_4_@C‐t electrodes exhibit lower internal and interfacial resistances than pure NiCo_2_S_4_. H‐NiCo_2_S_4_@C‐40 shows the lowest internal and interfacial resistances (0.76 and 0.44 Ω, respectively), demonstrating that the optimal carbon coating significantly reduces the charge transfer barrier and improves reaction kinetics (Table S7). The improved internal and interfacial resistances are attributed to the fact that the electron transport capacity of electrode and the affinity between the hybrid composite and electrolyte ion are effectively enhanced, which fundamentally enhance the electrochemical performance of the electrode [75]. Figure 4d and Figure S20 show the CV and GCD curves of optimized H‐NiCo_2_S_4_@C‐40 with different scan rates and various current densities. The pair of redox peaks and charge/discharge plateaus in the CV and GCD curves can be associated with M‐S‐OH and M‐S (M refers to the Ni and Co) reversible Faraday reaction on the surface [40]. Moreover, the basic shape of CV curves maintains well with the increase of scan rate, indicating an ideal rate capability [76]. Figures S21–S23 show the GCD curves of NiCo_2_S_4_, H‐NiCo_2_S_4_@C‐20, and H‐NiCo_2_S_4_@C‐60 electrodes at different current densities from 1 to 100 A g^−1^. The relationship between specific capacitance and current density is shown in a radar map (Figure 4e). The highest specific capacitance of 1501 F g^−1^ (Table S2 shows the mass loading of H‐NiCo_2_S_4_@C‐t electrodes) is calculated for H‐NiCo_2_S_4_@C‐40 electrode at the current density of 1 A g^−1^, which is about 1.5 times to that of pure NiCo_2_S_4_ electrode (1028 F g^−1^). Increasing the current density 20‐fold up to 20 A g^−1^, 84% of initial capacitance is retained, verifying the excellent rate capability of current H‐NiCo_2_S_4_@C‐40 electrode. Moreover, the obtained *b*‐values is 0.67 and 0.74 for anodic and cathodic peaks, respectively (Figure S24), suggesting a dominant diffusion‐controlled process for charge storage [11]. The surface capacitive contribution for electrode increases with the increase in scan rate (from 2 to 50 mV s^−1^) and finally reached value of 92% at 50 mV s^−1^ (Figures S25–S27). At low scan rate, the charge storage is dominated by diffusion‐controlled processes due to the sufficient Faraday reaction. As the scan rate increase, the surface capacitive behavior takes the lead. The cycling stability of H‐NiCo_2_S_4_@C‐40 electrode is evaluated by conducting consecutive GCD tests at a current density of 40 A g^−1^ (Figure 4f). A capacity retention of 90.8% is achieved after 10 000 cycles, which is much higher than those in several latest reports on NiCo_2_S_4_‐based electrodes (Table S8). Post‐cycling SEM (Figure S28) observations confirm that the 3D structure of H‐NiCo_2_S_4_‐40 is well maintained due to the carbon encapsulation, whereas the pristine NiCo_2_S_4_ electrode suffers from severe structural collapse.

The self‐discharge performance of NiCo_2_S_4_ and H‐NiCo_2_S_4_@C‐40 electrodes are exhibited by open‐circuit potential and leakage current measurements (Figure 4g–i). Charging both electrodes to 0.5 V at 1 A g^−1^ and holding the potential for 2 h, the potential of H‐NiCo_2_S_4_@C‐40 drops from 0.5 to 0.25 V over of H‐NiCo_2_S_4_@C‐40 electrode about 41 993s, which longer than the NiCo_2_S_4_ (21711s), showing H‐NiCo_2_S_4_@C‐40 electrode can significantly inhibit the self‐discharge rate. The inset of Figure 4g shows the leakage currents of pure NiCo_2_S_4_ and H‐NiCo_2_S_4_@C‐40 electrode, which was closely related to the self‐discharge performance. The steady‐state leakage current of H‐NiCo_2_S_4_@C‐40 electrode (95 *µA*) is much lower than that of NiCo_2_S_4_ (780 *µA*), corresponding to the result of the decay of open‐circuit potential. Figure 4h,i shows the decay of open‐circuit potential of both electrodes after charging at different current densities without holding process. As mentioned above (Figure 2h,i), the cumulative amount of ions near the surface caused by current density effects not only on self‐discharge but also on the initial potential. A low current density corresponds to a low self‐discharge rate and a high initial potential. Attentively, the self‐discharge and initial potential of H‐NiCo_2_S_4_@C‐40 electrode are optimized compared with NiCo_2_S_4_ electrode, due to the strengthen interfacial affinity between its clear carbon surface and OH^−^ [59].

**FIGURE 5:**
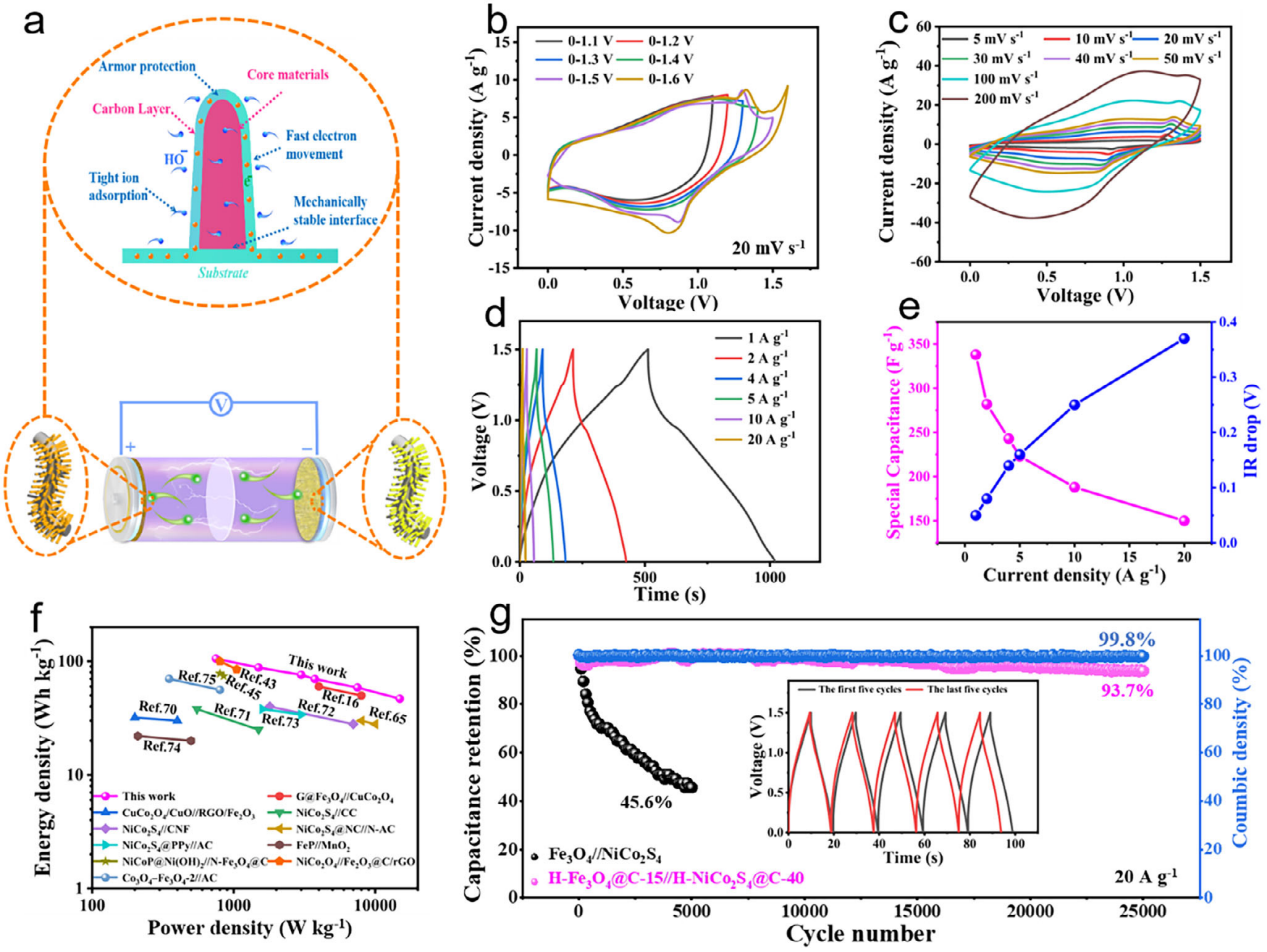
(a) Schematic illustration of the ASC (H‐Fe_3_O_4_@C‐15//H‐NiCo_2_S_4_@C‐40). Electrochemical performances of the as‐prepared H‐Fe_3_O_4_@C‐15//H‐NiCo_2_S_4_@C‐40. (b) CV curves obtained in different voltage windows at 20 mV s^−1^. (c, d) CV and GCD curves at various scan rates and current densities. (e) Specific capacitance and IR drop versus current densities. (f) Ragone plot related to energy and power densities of our cell and similar devices ever reported. (g) Cycling stability and corresponding Coulombic efficiency of our cell and Fe_3_O_4_//NiCo_2_S_4_, and the inset shows the GCD curves of our ASC with the first five cycles and the last five cycles.

Evaluating the as‐fabricated electrode materials for practical application, an ASC is built by employing the H‐Fe_3_O_4_@C‐15 as negative electrode and H‐NiCo_2_S_4_@C‐40 as positive electrode in 6.0 m KOH with one piece of cellulose paper as separator (H‐Fe_3_O_4_@C‐15//H‐NiCo_2_S_4_@C‐40), as shown in Figure 5a. The advanced nature of current ASC mainly stems from the configuration of the electrode as well as the reasonable design of active materials. With respect to the configuration of electrode, the asymmetric structure constructed with distinct positive and negative electrodes increases the cell voltage window, enabling a high energy density. The active materials (H‐Fe_3_O_4_@C‐15 and H‐NiCo_2_S_4_@C‐40) are directly grown on the highly conductive CF substrate. This mechanically stable free‐standing structure not only provides reliable anchoring for active material on current collector but also facilitates the electron transfer, thereby preventing the material shedding and lowering the internal resistance [38]. In terms of the optimized active materials, 1D linear nanostructure enhances electrolyte accessibility to the active material surface, favoring the full utilization of material. The conductive carbon shell allows rapid electron transport and electrolyte penetration/diffusion, promoting electrochemical kinetics. This tough carbon armor can also restrain the furious volume change of the core during the charge–discharge process, resisting the collapse of active material. Furthermore, thanks to the clean carbon shell surface, the tight‐bonding model between the carbon shell and ions, combined with fewer spontaneous Faraday reactions, collectively contributes to the suppressing self‐discharge in the open‐circuit condition [39].

Benefiting from the matched potential windows of H‐Fe_3_O_4_@C‐15 and H‐NiCo_2_S_4_@C‐40 (Figure S29), the maximum stable operating voltage of the assembled ASC is ascertained as 1.5 V (Figure 5b). The CV profiles of ASC at different scan rates show paired redox peaks (Figure 5c), and the small shift in the peaks with increasing scan rate suggests rapid reaction kinetics. Figure 5d shows nearly symmetric GCD curves at different current densities, implying a highly reversible behavior of device. The calculated specific capacitance is 338 F g^−1^ at a current density of 1 A g^−1^ (Figure 5e), which outperforms those of previously developed similar devices (Table S9). Moreover, the voltage drop (IR drop) of this ASC is relatively low (0.37 V at 20 A g^−1^), suggesting the fast ion transmission [15]. The Nyquist plot (Figure S30) shows a low internal resistance (1.69 Ω), and no obvious semicircle is observed in the middle frequency region, hinting high electrical conductivity and rapid charge transport kinetics. For a clear comparison, the energy and power densities of current ASC device and those of similar reported systems are summarized in Table S10. Furthermore, our ASC device delivers a maximum energy density of 105.6 W h kg^−^^1^ at a power density of 749 W kg^−^^1^, which is superior to most of the previously reported similar devices (Figure 5f) [16, 49, 51, 72, 77, 78, 79, 80, 81, 82]. Even at a high power density of 14.9 kW kg^−1^, the energy density can also approach to 46.9 W h kg^−1^. In addition, an ASC with 6.0 m KOH containing 0.03 m K_3_Fe(CN)_6_ electrolyte also demonstrates highly reversible elecrochemical behavior (Figure S31a,b). The Nyquist plot (Figure S31c) shows a low internal resistance 1.72 Ω and the steep line in the low‐frequency range suggests the fast ion diffusion. Furthermore, the device delivers a maximum energy density of 110 Wh kg^−1^ at a power density of 751 W kg^−1^ (Figure S31d). This higher energy density is mainly attributed to the extra faradaic redox reactions provided by [Fe(CN)_6_]^3−^/[Fe(CN)_6_]^4−^ pairs in the electrolyte. It is worth noting that the device exhibits a power density of 15 kW kg^−1^ at the corresponding energy density of 9 Wh kg^−1^. Compared with the pure KOH system, the reduced power density is primarily ascribed to the kinetic limitations caused by the large ion size of the Fe(CN)_6_^3−^. Figure 5g presents the cycling stability and Coulombic efficient of H‐Fe_3_O_4_@C‐15//H‐NiCo_2_S_4_@C‐40 for 25 000 cycles and Fe_3_O_4_//NiCo_2_S_4_ for 5000 cycles at 20 A g^−1^, respectively. Both cells display Coulombic efficient close to 100%. Divaricately, the capacitance retention of H‐Fe_3_O_4_@C‐15//H‐NiCo_2_S_4_@C‐40 reaches a remarkable ∼93.7% after 25 000 cycles, which is much higher than that of Fe_3_O_4_//NiCo_2_S_4_ (45.6% after 5000 cycles) and other previously reported similar devices (Table S9). The GCD curves with the first five cycles and the last five cycles (the inset in Figure 5g) disclose negligible variations, verifying the outstanding long‐term stability of H‐Fe_3_O_4_@C‐15//H‐NiCo_2_S_4_@C‐40 for repetitive charge/discharge cycling. Additionally, the cycling stability of current device at a low current density of 5 A g^−^^1^ is performed to better assess the long‐term durability (Figure S32), showing the 97.1% capacitance retention after 10 000 cycles. Furthermore, the XPS spectra of two electrodes after long‐term cycling (Figure S33) show that there is no obvious shift in the characteristic peak positions, confirming the excellent compositional and structural stability of both composites during repeated cycling, which is the key to the excellent cycling reversibility and high capacitance retention of the device.

**FIGURE 6:**
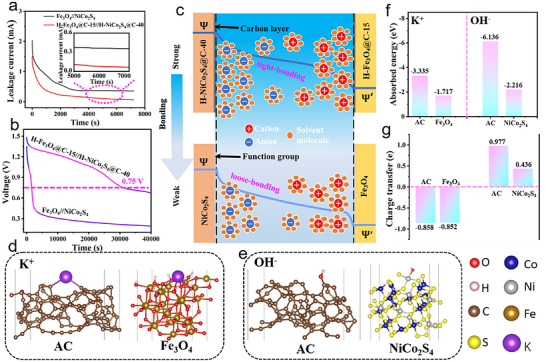
The self‐discharge performance of H‐Fe_3_O_4_@C‐15//H‐NiCo_2_S_4_@C‐40 and Fe_3_O_4_//NiCo_2_S_4_: (a) leakage current and (b) decay of open‐circuit voltage after holding voltage at 1.5 V for 2 h, (c) schematic illustration of the different bonding models. The adsorbed structures of (d) K^+^‐AC and K^+^‐Fe_3_O_4_ (e) OH^−^‐AC and OH^−^‐NiCo_2_S_4_, and the corresponding adsorption energies of (f) K^+^ and OH^−^ for the systems. (g) The charge transfer magnitudes relative to the structural surface.

Figure 6a,b presents the self‐discharge behaviors of H‐Fe_3_O_4_@C‐15//H‐NiCo_2_S_4_@C‐40 and Fe_3_O_4_//NiCo_2_S_4_, which were charged to 1.5 V and held at this voltage for 2 h at the current density of 1 A g^−1^. Evidently, the steady‐state leakage current of H‐Fe_3_O_4_@C‐15//H‐NiCo_2_S_4_@C‐40 (76 *µA*) is much lower than that of the Fe_3_O_4_//NiCo_2_S_4_ (370 *µA*). Additionally, the detected initial voltages of H‐Fe_3_O_4_@C‐15//H‐NiCo_2_S_4_@C‐40 and Fe_3_O_4_//NiCo_2_S_4_ are 1.48 and 1.34 V, respectively (Figure 6b). In view of charging continues under a constant voltage, the ions adsorbed on the surfaces of the positive and negative electrodes can be regarded as uniformly distributed [83]. The thin carbon shell containing fewer functional groups facilitates direct ion interaction during charging, thereby forming a tight‐bonding model; whereas, the abundance of function groups (e.g., originating from oxygen defects) on pure Fe_3_O_4_/NiCo_2_S_4_ increases the interaction distance, resulting in a loose‐bonding model, as illustrated in Figure 6c [58]. Therefore, the lower initial voltage at the beginning of the self‐discharge process for Fe_3_O_4_//NiCo_2_S_4_ should be attributed to the rapid desorption of some loose‐bonding ions. In the second stage of the voltage attenuation curves, H‐Fe_3_O_4_@C‐15//H‐NiCo_2_S_4_@C‐40 exhibits a more gradual voltage degradation than Fe_3_O_4_//NiCo_2_S_4_ (e.g., 1.32 to 0.76 V over 29470 s *vs* 1.04 to 0.39 V over 2720 s). This improved stability mainly because the carbon coating serving as solid–liquid interface inhibiting the continuous occurrence of side reactions on the electrode surface [59]. The voltage of H‐Fe_3_O_4_@C‐15//H‐NiCo_2_S_4_@C‐40 drops from 1.48 to 0.75 V over 31102 s, demonstrating a significantly lower self‐discharge rate compared to Fe_3_O_4_//NiCo_2_S_4_, which underwent the decay from 1.34 to 0.75 V in only 1607 s. The DFT calculations (see the details in Experimental Section, Supporting Information) are performance to further understand the changes in adsorption energy of Fe_3_O_4_, NiCo_2_S_4_, and their derivatives (using surface amorphous carbon (AC) as the object). Figure S34 and Figure 6d,e show the structural models of AC, Fe_3_O_4_, NiCo_2_S_4_, and corresponding ion adsorption structure models. To assess the stabilities of K‐AC, K‐Fe_3_O_4_, OH‐AC, and OH‐NiCo_2_S_4_, the absorption energy as calculated with *E*_ads_ = *E*_total_ − *E*_slab_ − *E*_iso_, where *E*_total_ and *E*_slab_ are the total energies of the surface slabs with and without the ions adsorbate, and *E*_iso_ is the isolate energy of K atom or OH group [84, 85]. As shown in Figure 6f, the adsorption energies of K‐AC (−3.335 eV) and OH‐AC (−6.136 eV) are higher than those of K‐Fe_3_O_4_ (−1.717 eV) and OH‐NiCo_2_S_4_ (−2.216 eV). According, the charge transfer analysis (Figure 6g) reveals that AC exhibits superior charge transfer capability with both ions (K^+^ and OH^−^) than Fe_3_O_4_ (−0.858 e *vs* −0.852 e with K^+^) and NiCo_2_S_4_ (0.977 e *vs* 0.436 e with OH^−^), which is directly responsible for its enhanced adsorption capacity [86]. The higher adsorption energy indicates a stronger affinity between the ions (K^+^ and OH^−^) and the carbon shell surface, leading to a more stable system and thus suppressing the self‐discharge process of H‐Fe_3_O_4_@C‐15//H‐NiCo_2_S_4_@C‐40.

## 3 Conclusions

In summary, the carbon coated H‐Fe_3_O_4_@C‐15 and H‐NiCo_2_S_4_@C‐40 electrodes are fabricated to construct a high‐performance ASC. The thickness and graphitization of surface carbon shell are optimized by the controllable electrodeposition and rapid Joule heating calcination. Benefiting from the asymmetric electrode configuration and the reasonable design of active materials (short‐range ordered carbon shells coating high‐capacity core pseudocapacitive materials), as‐assembled ASC exhibits a high energy density of 105.6 W h kg^−1^ at 749 W kg^−1^ and long‐term cycling stability (93.7% capacitance retention after 25 000 cycles). Moreover, the moderate self‐discharge behavior of H‐Fe_3_O_4_@C‐15//H‐NiCo_2_S_4_@C‐40 is also discovered (open‐circuit voltage drops from 1.48 to 0.75 V in 31102 s). Theoretically, the DFT results adequately uncover that the significantly improved self‐discharge performance should originate from the increased adsorption energy between the electrode and the electrolyte ions. Meaningfully, the controllable carbon shell encapsulation strategy developed in this work represents a universal and feasible approach to effectively suppress self‐discharge and extend the cycle life of ASC.

## Funding

This work was supported by National Natural Science Foundation of China (NSFC, Grant Nos. 52202246, 52202245), Jiangsu Special Term Professor Program, Youth Talent Support Program of Jiangsu Association for Science and Technology, Beforehand research project of New materials computing research center (No. 3700–32601).

## Conflicts of Interest

The authors declare no conflicts of interest.

## Date Availability Statement

The data that support the findings of this study are available from the corresponding author upon reasonable request.

## Supporting information




**Supporting File**: advs76184‐sup‐0001‐SuppMat.docx.
