# Supplementary material for: Controllable Carbon Shell Encapsulation via Rapid Joule Heating Calcination for High‐Performance Asymmetric Supercapacitor With Suppressed Self‐Discharge and Robust Cycling Stability
Source: Adv Sci (Weinh). 2026 Jun 18:e76184. Online ahead of print. doi: 10.1002/advs.76184 (PMC13336865; doi:10.1002/advs.76184)
Supplement: Supplementary file 1 — Supporting File: advs76184‐sup‐0001‐SuppMat.docx. [file ADVS-9999-e76184-s001.docx]

Supporting Information

Controllable Carbon Shell Encapsulation via Rapid Joule Heating Calcination for High-performance Asymmetric Supercapacitor with Suppressed Self-discharge and Robust Cycling Stability

*Qiang Zhou, Zheng Yang, Zhen Cao, Xulan Zheng, Xiao Yan, Yunsong Li, Xinsheng Zhao,* Yuxiao Lin,* Xiaoxiao Li,**

Experimental Section

*Pretreatment of carbon fiber (CF)*: The pieces of CF (1×2 cm^2^) were immersed into the 6.0 M nitric acid and subjected to hydrothermal treatment at 100 °C for 6 h. Then, these pieces were washed several times with ethanol and deionized water (DI) until pH = 7, followed by drying at 70 °C for 12 h.

*Fabrication of Fe_3_O_4_ nanorods on CF*: In a typical process, 4 mmol Fe(NO_3_)_3_∙9H_2_O, 4 mmol NH_4_F, and 20 mmol CO(NH_2_)_2_ with 60 ml DI were poured into the Teflon-lined autoclave under constant magnetic stirring 60 minutes. The processed CF was immersed into the above solution, maintained at 120 °C for 10 hours and then cooled to room temperature naturally to grow Fe_3_O_4_ precursors on the CF. Then, the CF was taken out and rinsed with DI water and ethanol to remove the other impurities. Finally, the precursors were transformed into Fe_3_O_4_ nanorods via the calcination at 350 °C for 1 h at a ramping rate of 2 °C min^-1^ under the atmosphere mixture of 95 vol.% Ar and 5 vol.% H_2_ at a flowing rate of 200 sccm.

*Fabrication of H-Fe_3_O_4_@C* *electrodes*: The preparation of the H-Fe_3_O_4_@C electrodes went through the processed of electrodeposition of polymer precursors, low temperature annealing and rapid Joule heating calcination. Firstly, the electrodeposition was performed using a solution of 0.15 g KCl and 0.4 mL pyrrole in 60 mL DI, with a three-electrode system: CF-supported Fe_3_O_4_ nanorods (working electrode), a platinum plate (counter electrode), and an Ag/AgCl electrode (reference electrode). And a constant potential deposition was conducted at 0.8 V for varied durations (10, 15, and 20 minutes). After that, the obtained products were calcined at 450 °C for 2 hours under N_2_ (70 sccm), and denoted as Fe_3_O_4_@C-t (t = 0, 10, 15, 20, referred to the time of electrodeposition reaction). Finally, these intermediates were subsequently subjected to a rapid Joule heating calcination at 800 °C for 5 s under Ar, producing the target products designated as H-Fe_3_O_4_@C-t. The mass loading of H-Fe_3_O_4_@C-t electrodes is shown in the following table:

**Table S1** The mass loading of H-Fe_3_O_4_@C-t electrodes versus the electrodeposition time.

| **Electrodeposition time (min)** | 0 | 10 | 15 | 20 |
| --- | --- | --- | --- | --- |
| **Mass loading**  **(mg cm^-2^)** | 0.8 | 1.3 | 1.5 | 1.9 |

*Preparation of NiCo_2_S_4_ Nanowires on CF*: NiCo_2_S_4_ nanowires grown on the CF were prepared by two-step hydrothermal route. Initially, the solution was achieved through mixing CoCl_2_∙6H_2_O (2.5 mmol), NiCl_2_∙6H_2_O (1.25 mmol), sodium dodecyl sulfate (SDS) (1 mmol) and urea (9 mmol) in 35 ml DI under vigorous magnetic stirring for 60 min, after which it was transferred into a 50 ml Teflon-lined autoclave. The processed CF was immersed into the above solution, maintained at 100 °C for 5 hours and then cooled to room temperature naturally to grow Ni-Co precursors (NiCo_2_(OH)_6_). Subsequently, after being rinsed with DI and ethanol, the CF with the Ni-Co precursors was subjected to a hydrothermal sulfidation reaction in 70 mL aqueous solution of 0.7 g Na_2_S at 120 °C for 7 hours, resulting in the formation of NiCo_2_S_4_ nanowires.

*Preparation of H-NiCo_2_S_4_@C electrodes*: The preparation process of H-NiCo_2_S_4_@C electrodes is similar to that of H-Fe_3_O_4_@C electrodes. Firstly, the electrodeposition was performed using a solution of 0.15 g KCl and 0.3 ml pyrrole in 100 mL DI, with a three-electrode system: CF-supported NiCo_2_S_4_ nanowires (working electrode), a platinum plate (counter electrode), and an Ag/AgCl electrode (reference electrode). And a constant potential deposition was conducted at 0.8 V for varied durations (20, 40, and 60 s). After that, the obtained products were calcined at 400 °C for 2 hours under N_2_ (70 sccm), and denoted as NiCo_2_S_4_@C-t (t = 0, 20, 40, 60, referred to the time of electrodeposition reaction). Finally, these intermediates were subsequently subjected to a rapid Joule heating calcination at 700 °C for 4 s under Ar, producing the target products designated as H-NiCo_2_S_4_@C-t. The mass loading of H-NiCo_2_S_4_@C-t electrodes is shown in the following table:

**Table S2** The mass loading of H-NiCo_2_S_4_@C-t electrodes versus the electrodeposition time.

| **Electrodeposition time**  **(s)** | 0 | 20 | 40 | 60 |
| --- | --- | --- | --- | --- |
| **Mass loading**  **(mg cm^-2^)** | 1.06 | 1 | 1.1 | 1.2 |

*Microstructural characterization*: The as-prepared samples were characterized by the field emission scanning electron microscopy (FE-SEM, SU-8010, Hitachi, Japan), high resolution transmission electron microscope (HRTEM, JEM-2100F, JEOL, Japan) equipped with energy-dispersive X-ray (EDX, Quantax-STEM, Bruker, Germany), X-ray diffraction (XRD, D8 Advance, Bruck, Germany), Raman spectrometry (InVia, Renishaw, U.K.) and X-ray photoelectron spectroscopy (XPS, AXIS ULTRA DLD, Shimadzu, Kyoto, Japan).

*Electrochemical measurement*: The electrochemical performances of the single electrode were investigated under a standard three-electrode configuration. The Fe_3_O_4_, Fe_3_O_4_@C-t, H-Fe_3_O_4_@C-t, NiCo_2_S_4_, NiCo_2_S_4_@C-t, and H-NiCo_2_S_4_@C-t were used as working electrodes. Pt foil, and Hg/HgO were used as the counter, and reference electrodes, respectively. The asymmetric coin cells (CR2032) were assembled by employing the H-Fe_3_O_4_@C-15 as negative electrode, H-NiCo_2_S_4_@C-40 as positive electrode and cellulose paper as separator. Based on the charge balance theory,^[1]^ the mass ratio of the positive and negative electrode active materials was determined to be 2:3. All electrochemical tests including cyclic voltammetry (CV), galvanostatic charging/discharging (GCD) and electrochemical impedance spectroscopy (EIS, the frequency range is 10^-2^ to 10^5^ Hz with a 5mV peak voltage for the open-circuit potential) were performed on an electrochemical station (CS2350M, CORRTEST, China) in 6.0 M KOH aqueous electrolyte at room temperature. For comparison, another asymmetric coin cell was assembled and tested in the same way using an electrolyte consisting of 0.03 M K_3_Fe(CN)_6_ and 6 M KOH.

The specific capacitance of the electrode in three-electrode system was calculated based on the GCD curve, according to Equation (1):

$\text{C}_{1}=\frac{I\Delta t}{m\Delta V}$ (F g^-1^) (1)

The specific capacitance of the as-assembled ASC was obtained from the GCD curve, according to Equation (2):

$C_{s}=\frac{I\Delta t}{M\Delta V}$ (F g^-1^) (2)

The energy density (*E*) and power density (*P*) of the ASC device were calculated by the equations (3) and (4), respectively:

$E=\frac{1}{2}\times C_{s}\times\frac{\left(\Delta V\right)^{2}}{3600}$ (W h kg^-1^) (3)

$P=\frac{E}{\Delta t}\times3600$ (W kg^-1^) (4)

where *I* is the discharge current (A), Δ*t* is the discharge time (s), *m* is the mass of active materials in single electrode (g), and Δ*V* is the voltage window of the discharging (V), *M* is the total mass of both positive and negative electrodes.

*Density Functional Theory (DFT) Computations*: All calculations were performed based on plane-wave DFT using the Vienna Ab initio Simulation Package (VASP).^[2]^ The projector augmented wave (PAW) method was employed to describe the interactions between core and valence electrons,^[3]^ while the generalized gradient approximation (GGA) with the Perdew–Burke–Ernzerhof (PBE) functional was used for the exchange-correlation energy.^[4]^ To accurately account for intermolecular dispersion interactions, the DFT-D3 correction with Becke-Johnson damping was applied.^[5]^ Spin polarizations were also included and the cutoff energies were 500 eV.

Isolated K atom and OH group were placed in a cubic simulation box of 10×10×10 Å^3^ for geometry optimization. The K-mesh was 1×1×1 based on the gamma center. The convergence criteria for energy and force were 1.0×10^-6^ eV and 0.01 eV/Å, respectively. The surface slabs of Fe_3_O_4_ (100) and NiCo_2_S_4_ (311) were cleaved based on the optimized bulk structures with supercell sizes of 2×2 and 1×2. Each surface slab contains 4 layers and a vacuum thickness larger than 15 Å. During the geometry optimization, only the top 2 layers and the adsorbate were allowed to move, while the bottom 2 layers were fixed. For Fe_3_O_4_ (100) surface slabs, H atoms were also added to eliminate the artificial influence of dangling O bonds. To get the bulk structure of amorphous carbon (AC), ab initio molecular dynamics (AIMD) simulation under the ensemble of constant volume and temperature (NVT) was performed based on the 4×3×2 supercell of optimized graphite bulk structure. The AIMD simulation consisted of a stepwise heating stage from 0 K to 9000 K for 50 ps with the timestep of 2 fs and an equilibration stage at 9000 K for 10 ps with the timestep of 1 fs, in which the temperature was controlled by the Nosé–Hoover thermostat.^[6]^ After that, the surface slab was cleaved and optimized. The structural parameters, lattice constants, and K-meshes are provided in Table S11.

The adsorption energy (Eₐ_ds_) of atoms was calculated using Equation (5), where E_total_ and E_slab_ are the total energies of the surface slabs with and without the adsorbate, respectively. E_iso_ is the energy of the isolate adsorbate, namely, the K atom or OH group.^[7,8]^

E_ads_ = E_total_ − E_slab_ − E_iso_ (5)


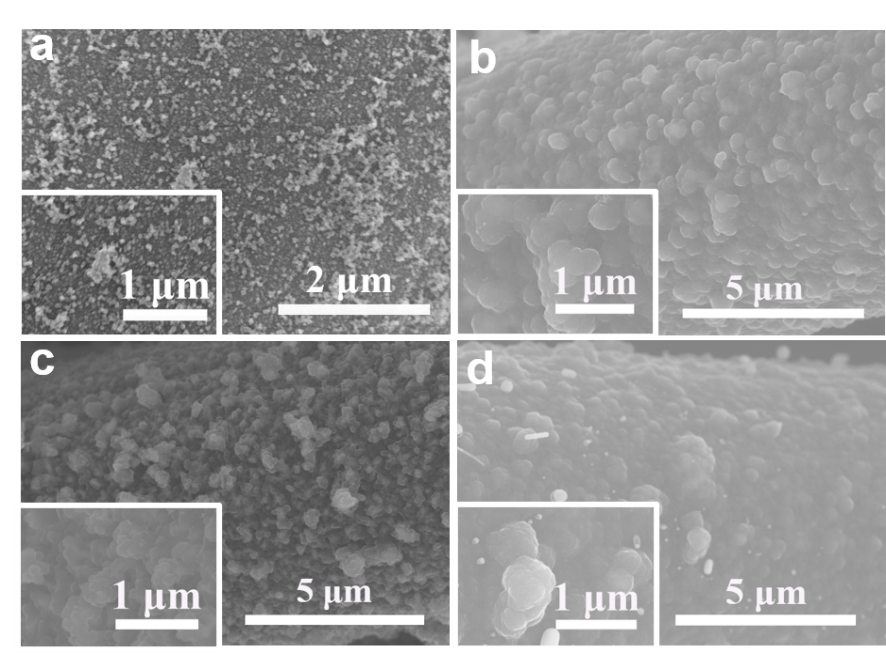


**Figure S1.** SEM images of (a) Fe_3_O_4_, (b) H-Fe_3_O_4_@C-10, (c) H-Fe_3_O_4_@C-15, (d) H-Fe_3_O_4_@C-20.


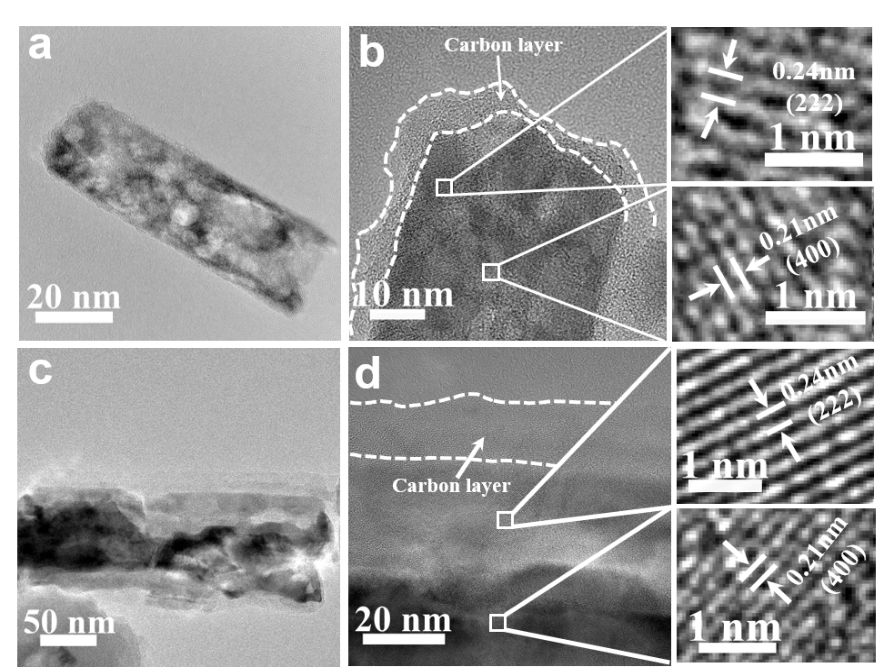


**Figure S2.** Typical TEM and HRTEM images of H-Fe_3_O_4_@C-10 (a and b) and H-Fe_3_O_4_@C-20 (c and d).


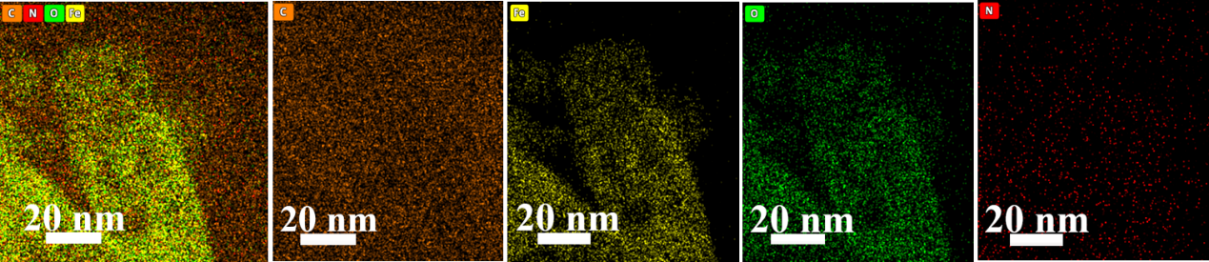


**Figure S3** The EDX mappings of the H-Fe_3_O_4_@C-15.

**Table S3** The atomic concentrations of Fe_3_O_4_ and H-Fe_3_O_4_@C-15 samples.

| Sample | Fe (At%) | O (At%) | N (At%) | C (At%) |
| --- | --- | --- | --- | --- |
| Fe_3_O_4_ | 14.40 | 22.32 | 1.04 | 62.24 |
| H-Fe_3_O_4_@C-15 | 2.28 | 3.13 | 8.28 | 86.31 |


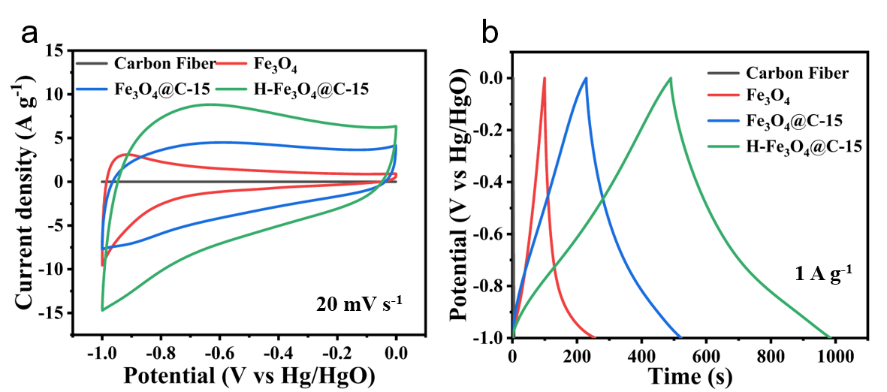


**Figure S4.** The electrochemical performance of CF, Fe_3_O_4_, Fe_3_O_4_@C-15 and H-Fe_3_O_4_@C-15 electrodes: (a) CV curves at a scan rate of 20 mV s^-1^ and (b) GCD curves at a current density of 1 A g^-1^.


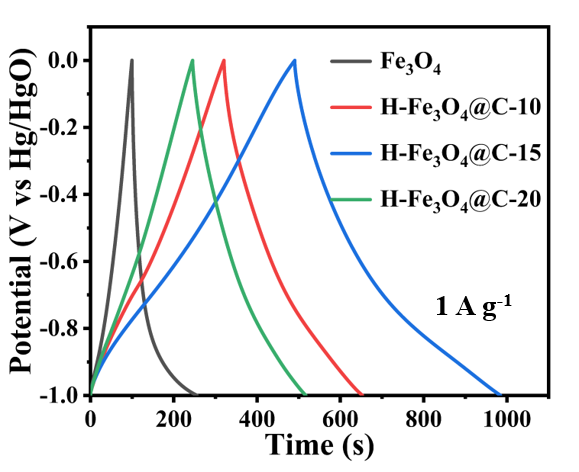


**Figure S5.** GCD curves of Fe_3_O_4_ and H-Fe_3_O_4_@C-t electrodes at a constant current density of 1 A g^-1^.

**Table S4** Internal resistance and interfacial resistance of the Fe_3_O_4_ and H-Fe_3_O_4_@C-t electrodes.

|  | Fe_3_O_4_ | H-Fe_3_O_4_@C-10 | H-Fe_3_O_4_@C-15 | H-Fe_3_O_4_@C-20 |
| --- | --- | --- | --- | --- |
| internal resistances (Ω) | 2.16 | 0.89 | 0.82 | 1.04 |
| interfacial resistances (Ω) | 1.8 | 1.14 | 0.68 | 0.86 |


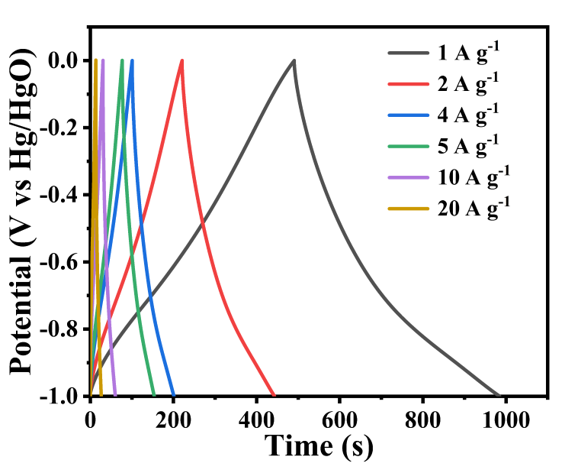


**Figure S6.** GCD curves of H-Fe_3_O_4_@C-15 electrode at different current densities.


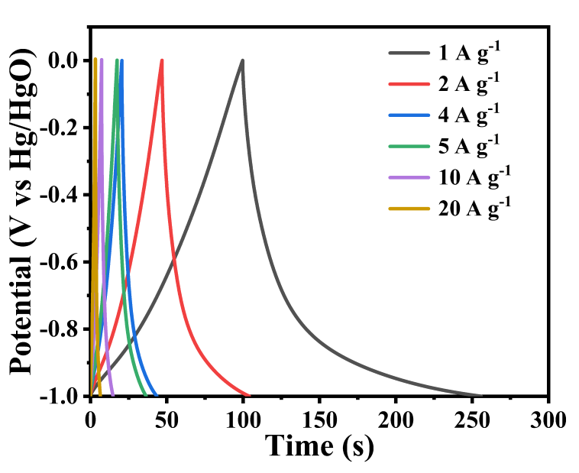


**Figure S7.** GCD curves of Fe_3_O_4_ electrode at different current densities.


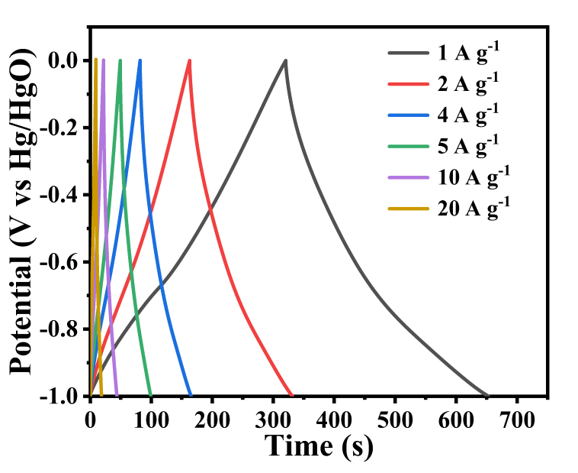


**Figure S8.** GCD curves of H-Fe_3_O_4_@C-10 electrode at different current densities.


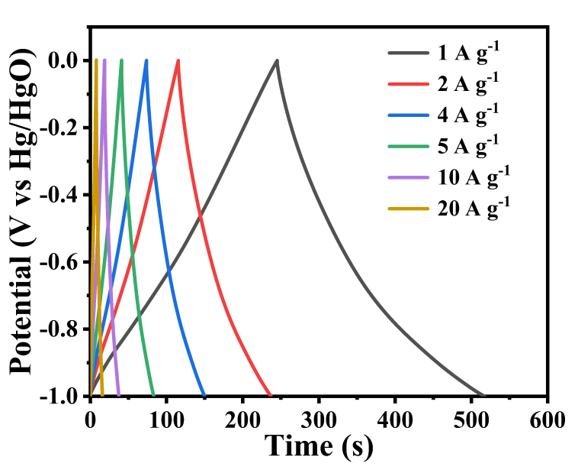


**Figure S9.** GCD curves of H-Fe_3_O_4_@C-20 electrode at different current densities.


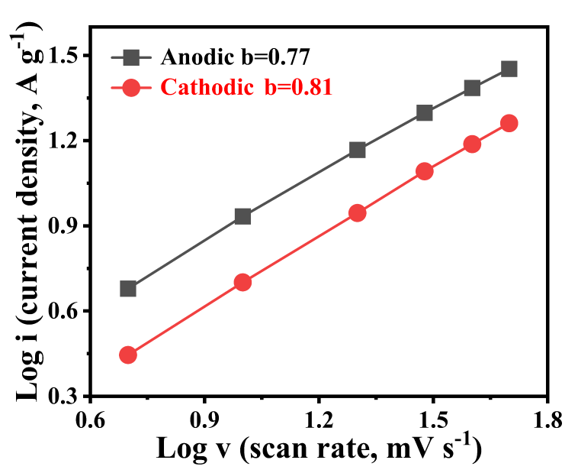


**Figure S10.** Plots of log(*i*) versus log(*v*) calculated from CV curves of H-Fe_3_O_4_@C-15.

The capacitive and diffusion behavior of H-Fe_3_O_4_@C-15 electrode is distinguished by analyzing the relationship between current density (i) and scan rate (*v*) according to the power law: *i* = *a v*^b^ and log(*i*) = *log*(*a*) + *b* log(*v*).^[9,10]^ Where, *a* and *b* (the fitting line of log(*i*) *vs* log(*v*)) are regarded as constants. The calculated *b* values of anodic and cathodic peaks are 0.77 and 0.81, suggesting that the mechanism of energy storage of H-Fe_3_O_4_@C-15 electrode is that the diffusion control and capacitance control coexist and cooperate with each other.^[11,12]^


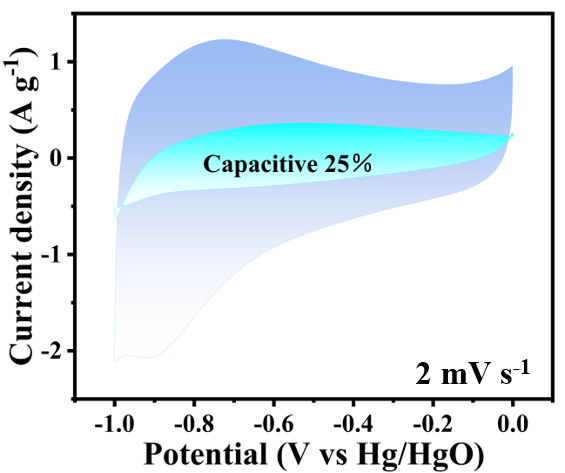


**Figure S11.** Capacitive contribution at 2 mV s^-1^ rates for the as-prepared H-Fe_3_O_4_@C-15 electrode.


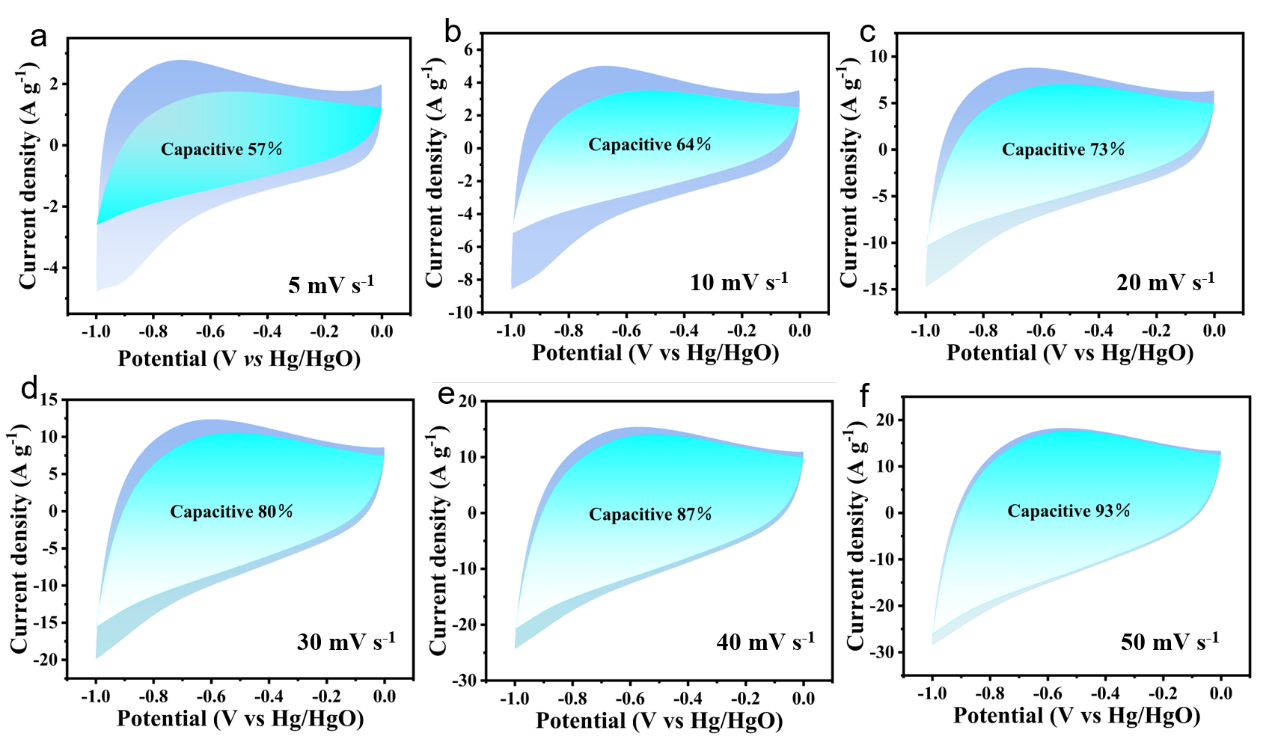


**Figure S12.** (a-f) Capacitive contribution at different scan rates for the as-prepared H-Fe_3_O_4_@C-15 electrode.

The quantification of the capacitive contribution to the total capacity is analyzed on the basis of the equation: *I*(*V*) = *k*_1_*v* + *k*_2_*v*^1/2^.^[13]^ Where, the i(*V*) is the total current at a constant potential *V*, *k*_1_ and *k*_2_ are constants, *k*_1_*v* and *k*_2_*v*^1/2^ are the current of contributions of capacitive and diffusion-controlled process, respectively.


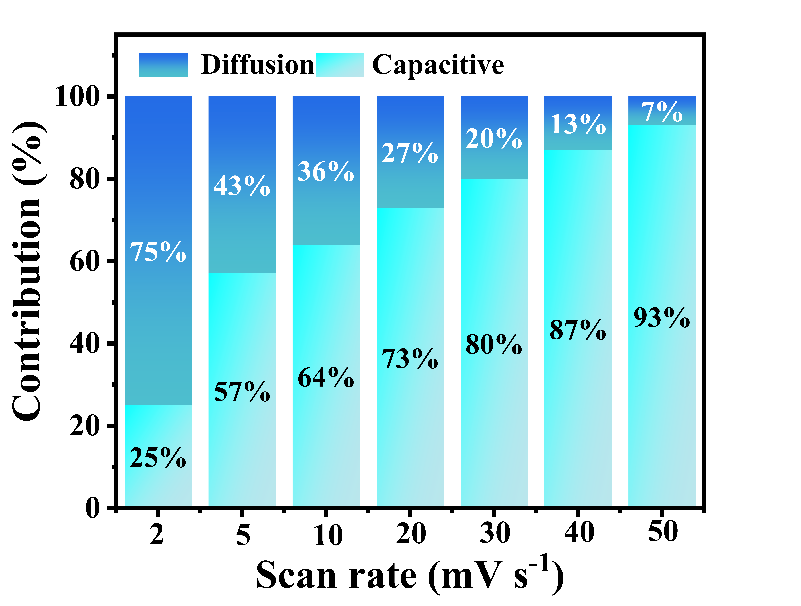


**Figure S13.** Contribution ratios of diffusion-controlled and surface capacitive charge of H-Fe_3_O_4_@C-15 at different scan rates.


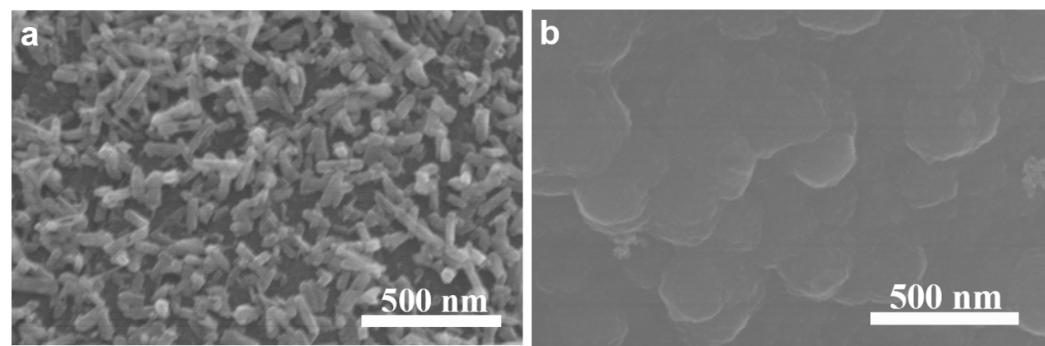


**Figure S14.** SEM images of (a) Fe_3_O_4_ after 2000 cycles and (b) H-Fe_3_O_4_ @C-15 after 20000 cycles, respectively.

**Table S5** Comparison of the electrochemical properties of the as-fabricated H-Fe_3_O_4_@C-15 with previously reported Fe_3_O_4_-based electrodes.

| **Materials** | **Electrolyte** | **Specific capacitance** | **Stability** | **Ref.** |
| --- | --- | --- | --- | --- |
| **H-Fe_3_O_4_@C-15** | **6 M KOH** | **493 F g^-1^ at 1 A g^-1^** | **99.8% for 20000** **cycles** **at 20 A g^-1^** | **This work** |
| Fe_2_O_3_@FeSe_2_ | 1 M Na_2_SO_4_ | 526.3 mF cm^-2^ at 1 mA cm^-2^ | 80.3% for 4000 cycles at 10 mA cm^-2^ | [14] |
| Fe_3_O_4_@ppy | 1M H_2_SO_4_ | 290.2 F g^-1^ at 1 A g^-1^ | - | [15] |
| Fe_3_O_4_@Carbon Nanosheets | 6 M KOH | 586 F/g at 0.5 A g^-1^ | - | [16] |
| Fe_3_O_4_@C@PANi | 1M KOH | 420 F g^-1^ at 0.5 A g^-1^ | 82% for 5000 cycles at 10 A g^-1^ | [17] |
| Fe_3_O_4_@Fc-GO/PANI | 0.5 M NaOH | 640mAh g^-1^ at 1 A g^-1^. | 86.68% for 5000 CV cycles | [18] |
| Fe_3_O_4_@Bi_2_O_3_ | 1M KOH | 136.1 mAh g^-1^ at 1 A g^-1^. | 81% for 5000 cycles | [19] |
| Fe_3_O_4_@NG | 6 M KOH | 740 F g^-1^ at 1 A g^-1^ | 90.9% for 3000 cycles at 1 A g^-1^ | [20] |
| Fe_3_O_4_@PPy@MnO_2_ | 1 M KOH | 751F g^-1^ at 1 A/g | 87.0 % for 5000 cycles at 4 A/g | [21] |
| Fe_3_O_4_@TiO_2_@C | 1 M Na_2_SO_3_ | 304.1 mF cm^-2^ at 1 mA cm^-2^ | 90.7% for 10000 cycles at 5 mA cm^-2^ | [22] |
| Fe_3_O_4_@MoS_2_/rGO | 6 M KOH | 527 F g^-1^ at 0.5 mA cm^-2^ | 93% for 5000 cycles at 100 mV s^-1^ | [23] |
| Fe_3_O_4_@PDA | 1 M KOH | 715 F/g at 1 A/g | - | [24] |
| Fe_3_O_4_/rGo | 3 M KOH | 807 F g^-1^ at 1 A g^-1^, | 85.5% for 10000 cycles at 10 Ag^-1^ | [25] |
| CNTs/MoS_2_/Fe_3_O_4_ | 6 M KOH | 356 F g^-1^ at 200 mV s^-1^ | - | [26] |
| BC/Fe_3_O_4_@PPy | 1M H_2_SO_4_ | 232.9 F g^-1^ at 1 A g^-1^ | 81.4% for 3500 cycles at 0.05 V s^-1^ | [27] |


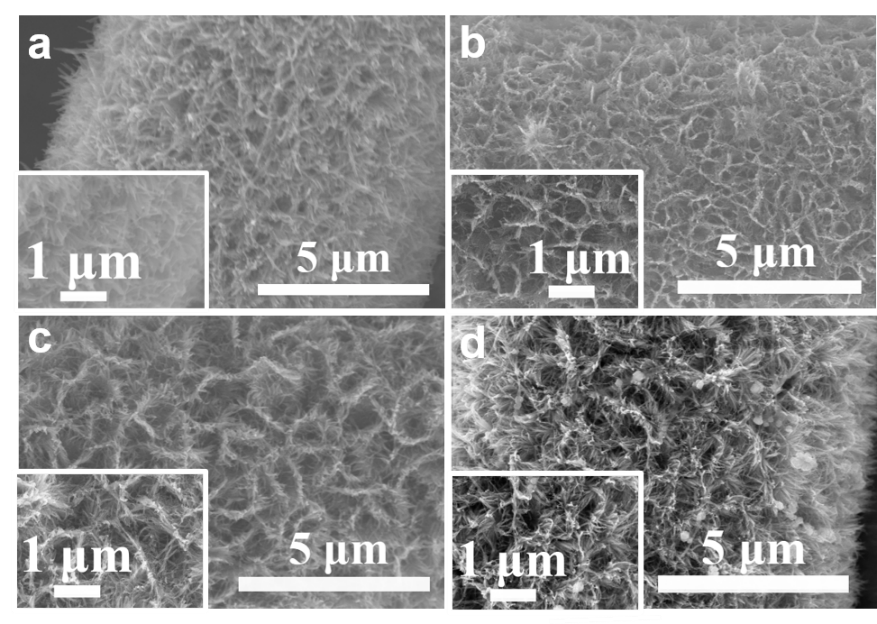


**Figure S15.** SEM images of the (a) NiCo_2_S_4_, (b) H-NiCo_2_S_4_@C-20, (c) H-NiCo_2_S_4_@C-40, (d) H-NiCo_2_S_4_@C-60.


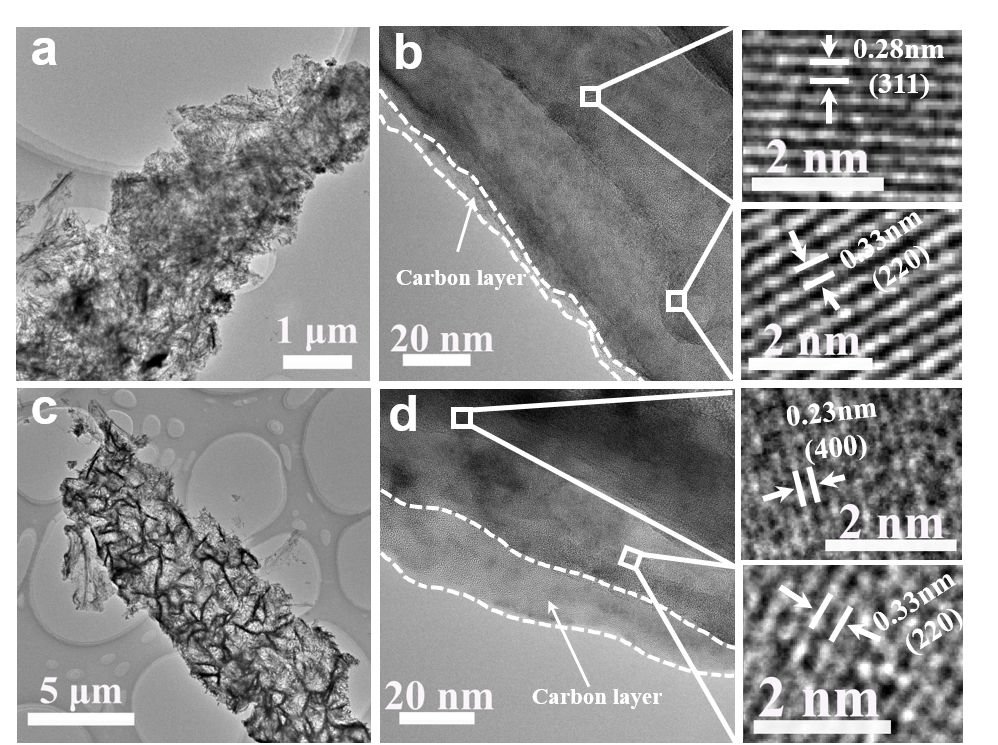


**Figure S16.** Typical TEM and HRTEM images of H-NiCo_2_S_4_@C-20 (a and b) and H-NiCo_2_S_4_@C-60 (c and d).


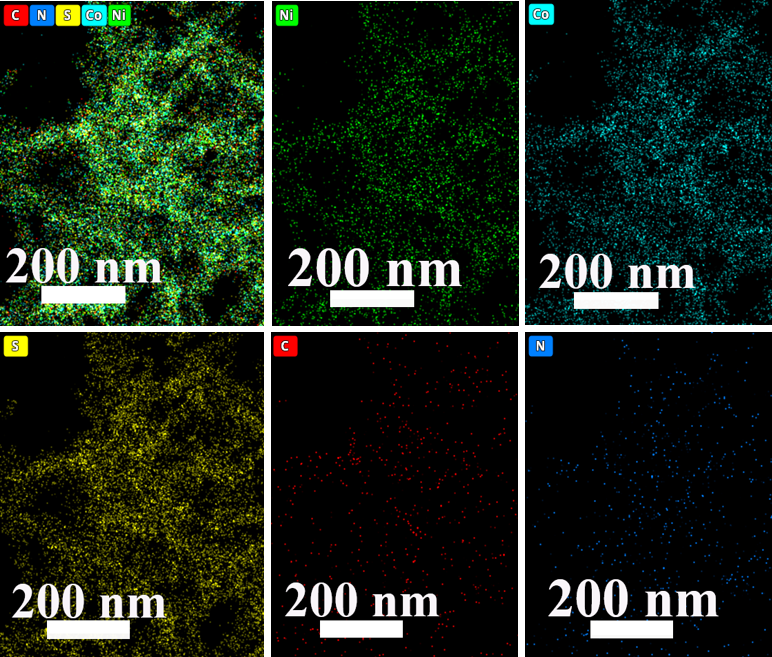


**Figure S17.** The EDX mappings of the H-NiCo_2_S_4_@C-40.

**Table S6** The atomic concentrations of NiCo_2_S_4_ and H-NiCo_2_S_4_@C-40 samples.

| Sample | Ni (At%) | Co (At%) | S (At%) | N (At%) | C (At%) |
| --- | --- | --- | --- | --- | --- |
| NiCo_2_S_4_ | 3.3 | 6.92 | 14.63 | 2.77 | 72.38 |
| H-NiCo_2_S_4_@C-40 | 3.46 | 5.38 | 9.05 | 2.52 | 79.59 |


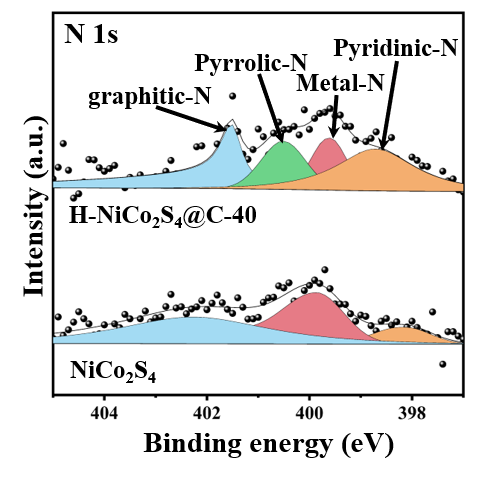


**Figure S18.** High-resolution XPS spectra of N 1s for NiCo_2_S_4_ and H-NiCo_2_S_4_@C-40.


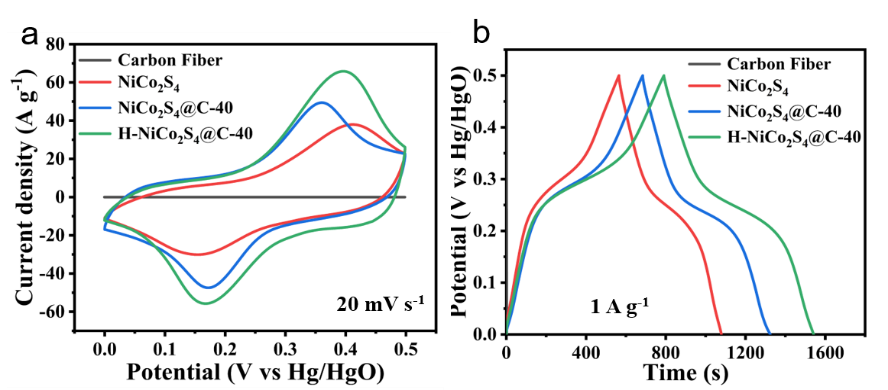


**Figure S19.** The electrochemical performance of CF, NiCo_2_S_4_, NiCo_2_S_4_@-40 and H-NiCo_2_S_4_@C-40 electrodes: (a) CV curves at a scan rate of 20 mV s^-1^ and (b) GCD curves at a current density of 1 A g^-1^.

**Table S7** Internal resistance and interfacial resistance of the NiCo_2_S_4_ and H-NiCo_2_S_4_@C-t electrodes.

|  | NiCo_2_S_4_ | H-NiCo_2_S_4_@C-20 | H-NiCo_2_S_4_@C-40 | H-NiCo_2_S_4_@C-60 |
| --- | --- | --- | --- | --- |
| internal resistances (Ω) | 1.2 | 0.98 | 0.76 | 0.93 |
| interfacial resistances (Ω) | 3.4 | 0.6 | 0.44 | 0.64 |


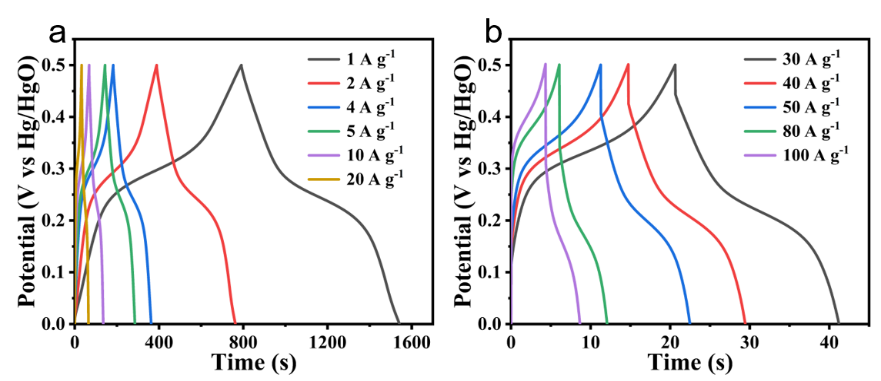


**Figure S20.** (a,b) GCD curves of H-NiCo_2_S_4_@C-40 electrode at different current densities**.**


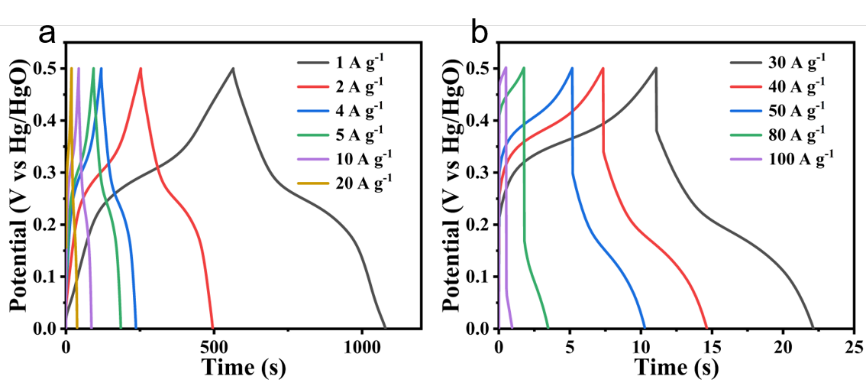


**Figure S21.** (a,b) GCD curves of NiCo_2_S_4_ electrode at different current densities.


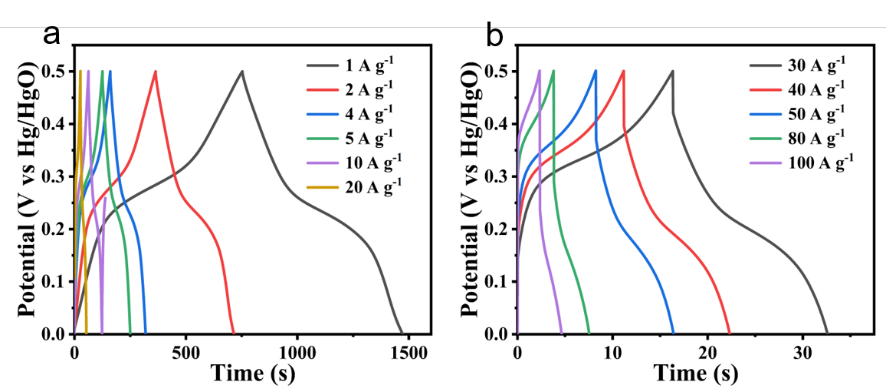


**Figure S22.** (a,b) GCD curves of H-NiCo_2_S_4_@C-20 electrode at different current densities.


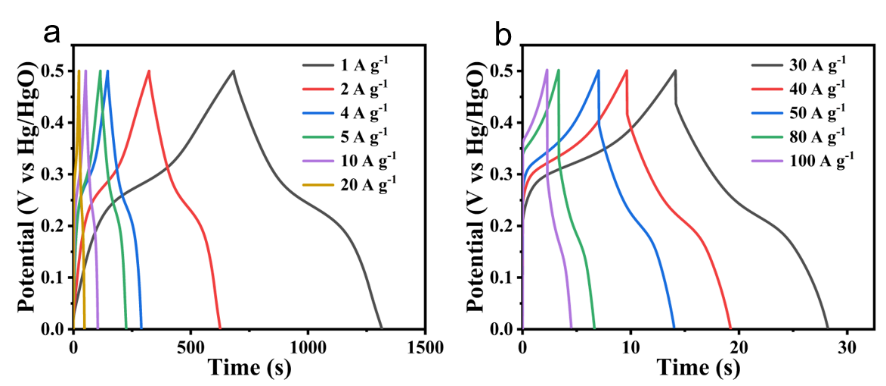


**Figure S23.** (a,b) GCD curves of H-NiCo_2_S_4_@C-60 electrode at different current densities.


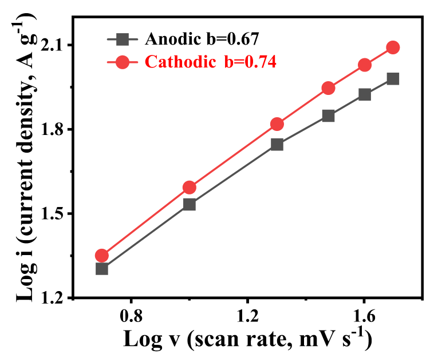


**Figure S24.** Plots of log(*i*) versus log(*v*) calculated from CV curves for the H-NiCo_2_S_4_@C-40 electrode.

The analytical method for the capacitive and diffusion behavior of H-NiCo_2_S_4_@C-40 electrode is the same as that for H-Fe_3_O_4_@C-15 electrode (Figure S10). The calculated *b* values of anodic and cathodic peaks are 0.67 and 0.74, suggesting that the mechanism of energy storage of H-NiCo_2_S_4_@C-40 electrode is that the diffusion control and capacitance control coexist and cooperate with each other.^[11,12]^


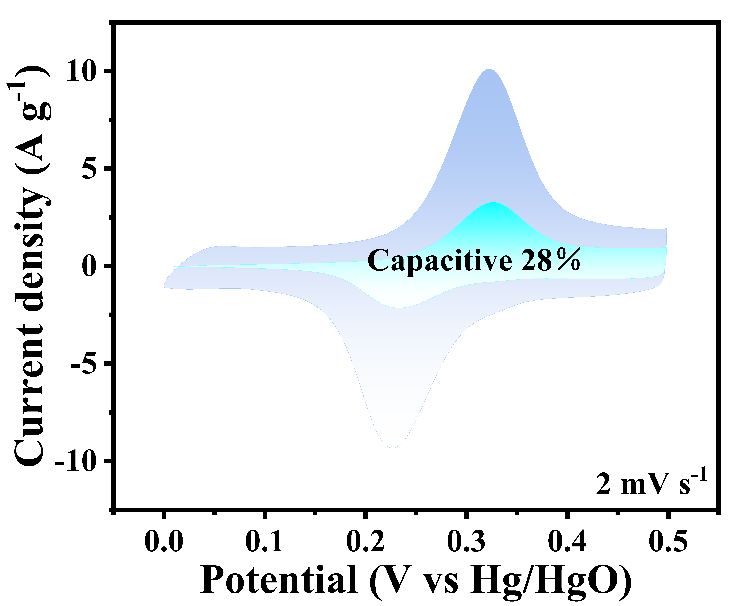


**Figure S25.** Capacitive contribution at 2 mV s^-1^ for the as-prepared H-NiCo_2_S_4_@C-40 electrode.


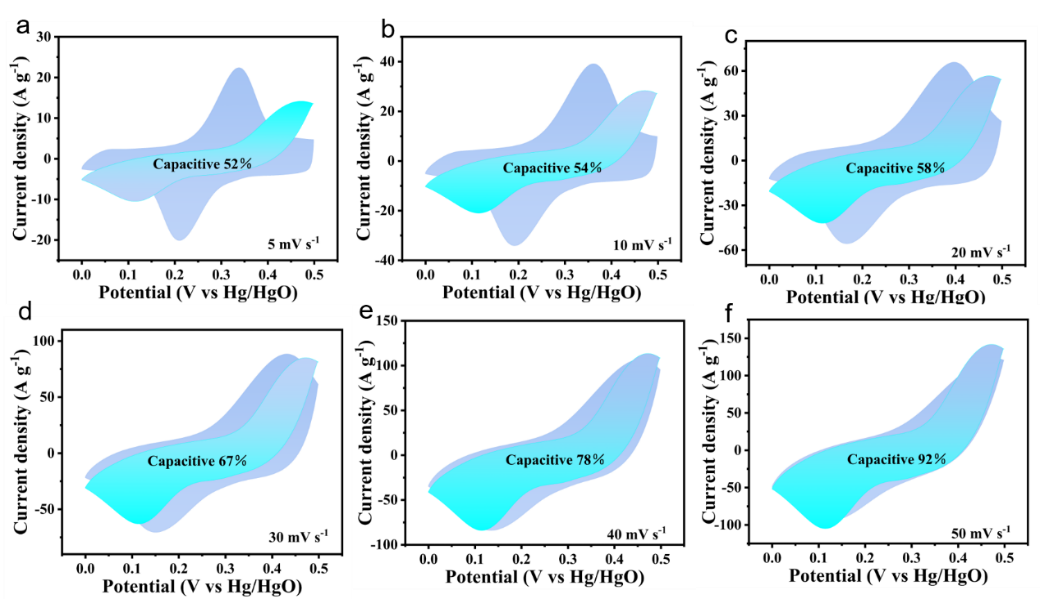


**Figure S26.** (a-f) Capacitive contribution at different scan rates for the as-prepared H-NiCo_2_S_4_@C-40 electrode.


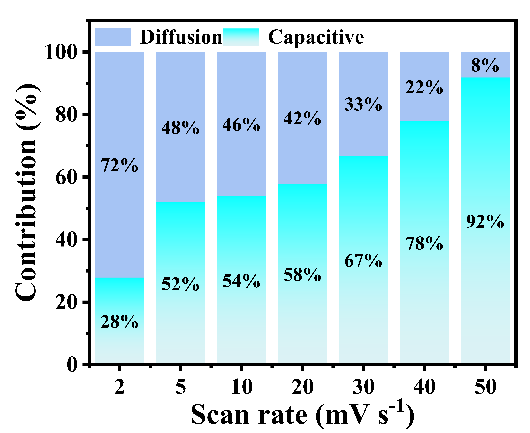


**Figure S27.** Contribution ratios of diffusion-controlled and surface capacitive charge of H-NiCo_2_S_4_@C-40 at different scan rates.

**Table S8** Comparison of the electrochemical properties of the as-fabricated H-NiCo_2_S_4_@C-40 with previously reported NiCo_2_S_4_-based electrodes.

| **Materials** | **Substrate** | **Specific capacitance** | **Stability** | **Ref.** |
| --- | --- | --- | --- | --- |
| **H-NiCo_2_S_4_@C-40** | **carbon fiber** | **1501 F g^-1^ at 1 A g^-1^** | **90.8% for 10000 cycles at 40 A g^-1^** | **This work** |
| NiCo_2_S_4_@polyaniline | Ni foam | 9.28 F cm^-2^ at 5 mA cm^-2^ | 61.64% for 5000 cycles at 20 mA cm^-2^ | [28] |
| NiFeP@NiCo_2_S_4_ | carbon cloth | 874.4 C g^-1^ at 1 A g^-1^ | 85.6% for 5000 cycles at 5 A g^-1^ | [29] |
| NiCo_2_S_4_@C | Ni foam | 1800 F/g at 1 A g^-1^ | 91% for 5000 cycles at 10 A g^-1^ | [30] |
| MgCo_2_O_4_@NiCo_2_S_4_ | Ni foam | 1255.1 C g^-1^ at 1 A g^-1^ | 78.07% for 2000 cycles at 5 A g^-1^ | [31] |
| Co_9_S_8_@NiCo_2_S_4_ | carbon cloth | 2020.6 F g^-1^ at 1 A g^-1^, | 89.8% for 10000 cycles at 10 A g^-1^ | [32] |
| NiCo_2_O_4_@NiCo_2_S_4_ | carbon cloth | 2243.0 F g^-1^ at 1 A g^-1^ | 83.29% for 5000 cycles at 10 A g^-1^ | [33] |
| NiCo_2_S_4_@NiMoO_4_ | Ni foam | 1102.5 F g^-1^ at 1 A g^-1^ | 72.7 % for 10000 cycles at 10 A g^-1^ | [34] |
| NiCo_2_S_4_@EGP | graphite paper | 1276 F g ^-1^ at 1 A g ^-1^ | 86 % for 5000 cycles at 20 A g^-1^ | [35] |
| NiCo_2_S_4_/ACT | carbon textile | 183.2 mA h g^-1^ at 2 mA cm^-2^ | 82.6 % for 1000 cycles at 20 mA cm^-2^ | [36] |
| NiCo_2_S_4_@NiCo_2_S_4_ | carbon cloth | 245 mA h cm^-2^ at 2 mA cm^-2^ | 86% for 3000 cycles at 10 mA cm^-2^ | [37] |
| ZnCo_2_O_4_@NiCo_2_S_4_@PPy | Ni Foam | 2507.0 F g^-1^ at 0.5 A g^-1^ | 83.2% for 5000 cycles at 10 A g^-1^ | [38] |
| MXene-NiCo_2_S_4_ | Ni Foam | 596.69 C g^-1^ at 1 A g^-1^ | 80.4% for 3000 cycles at 10 A g^-1^ | [39] |
| NiCoAl-LDH@NiCo_2_S_4_ | carbon cloth | 1775F g^-1^ at 1 A g^-1^ | 79.6% for 10000 cycles at 10 A g^-1^ | [40] |


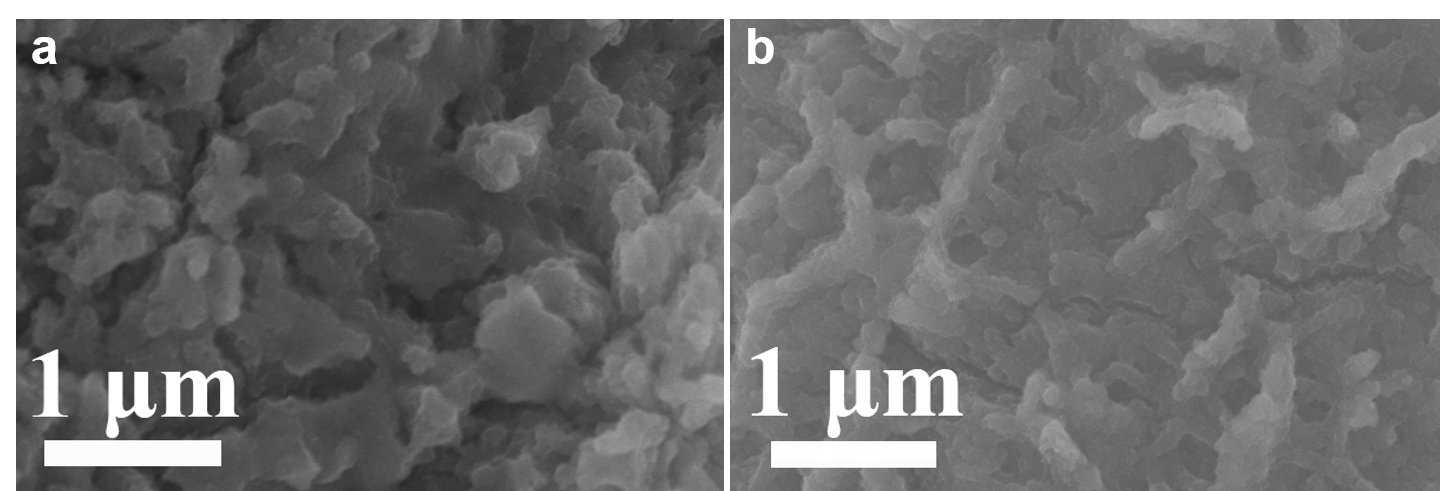


**Figure S28.** SEM images of (a) NiCo_2_S_4_ after 5000 cycles and (b) H-NiCo_2_S_4_@C-40 after 10000 cycles, respectively.


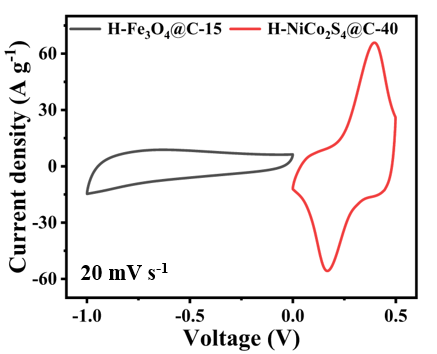


**Figure S29.** CV curves of H-Fe_3_O_4_@C-15 and H-NiCo_2_S_4_@C-40 at a scan rate of 20 mV s^-1^.

**Table S9** The comparison of our cell (H-Fe_3_O_4_@C-15//H-NiCo_2_S_4_@C-40) with previous reported similar devices.

| **Materials** | **Voltage window** | **Electrolyte** | **Specific capacitance** | **Stability** | **Ref.** |
| --- | --- | --- | --- | --- | --- |
| **H-NiCo_2_S_4_@C-40//H-Fe_3_O_4_@C-15** | **0-1.5V** | **6 M KOH** | **338 F g^-1^ at 1 A g^-1^** | **93.7% for 25000 cycles at 20 A g^-1^** | **This work** |
| CuCo_2_O_4_//G@Fe_3_O_4_ | 0-1.6V | 2 M KOH | 182 F g^-1^ at 2 A g^-1^ | 90.2% for 10000 cycles at 20 A g^-1^ | [41] |
| Fe_3_O_4_@MnO_2_//N-pC | 0-1.5V | 1 M KOH | 100 F g^-1^ at 1 A g^-1^ | 94.7% for 5000 cycles at 4 A g^-1^ | [24] |
| NiMoO_4_//Fe_3_O_4_@Bi_2_O_3_ | 0-1.65V | 1 M KOH | 47.3 mAh g^-1^ at 1 A g^-1^ | 98.6% for 2500 cycles at 2 A g^-1^ | [19] |
| SiC NWs@Fe_2_O_3_//SiC NWs@NiCo_2_O_4_/Ni(OH)_2_ | 0-1.75V | 2 M KOH | 242 F g^-1^ at 4 A g^-1^ | 86.6% for 5000 cycles at 9 A/g | [42] |
| Fe_3_O_4_@PPy@MnO_2_-2//AC | 0-1.5V | 1 M KOH | 228.3F g^-1^ at 1 A g^-1^ | 95.2 % for 5000 cycles at 5 A/g | [21] |
| NiCo_2_S_4_@PANI-5//AC | 0-1.6V | 3 M KOH | 2.84 F cm^-2^ at 5 mA cm^-2^ | 76.7% for 1000 cycles at 20 mA cm^-2^ | [28] |
| Co_3_S_4_@NiCo_2_S_4_//AC | 0-1.5V | 3 M KOH | 187 F g^-1^ at 1 A g^-1^ | 81.8% for 1000 cycles at 10 A/g | [43] |
| NiCoAl-LDH@NiCo_2_S_4_//AC | 0-1.5V | KOH/PVA | 106F g^-1^ at 1 A g^-1^ | 71.4% for 1000 cycles at 1 A/g | [40] |
| NiCo_2_S_4_@NiMoO_4_//AC | 0-1.6V | 3 M KOH | 234.2 F g^-1^ at 1 A g^-1^ | 75.0% for 10000 cycles at 10 A/g | [34] |
| NiCo_2_S_4_//Fe_2_O_3_ | 0-1.4V | KOH/PVA | 343.2 mF cm^-2^ at 1 mA cm^-2^ | 81.8% for 5000 cycles at 20 mA cm^-2^ | [44] |
| Co_9_S_8_@NiCo_2_S_4_//AC | 0-1.6V | KOH/PVA | 163.1 F g^-1^ at 1 A g^-1^ | 92.9% after 10000 at 10 A g^-1^ | [32] |
| NiFeP@NiCo_2_S_4_//OPC | 0-1.6V | 2 M KOH | 228.0 F g^-1^ at 0.5 A g^-1^ | 85.2 % for 10000 at 8 A g^-1^ | [29] |
| NiCo_2_O_4_@NiCo_2_S_4_//AC | 0-1.6V | KOH/PVA | 241.8 F g^-1^ at 1 A g^-1^ | 77.23 % after 10000 cycles at 10 A g^-1^ | [33] |
| MgCo_2_O_4_@NiCo_2_S_4_-2//AC | 0-1.6V | 6M KOH | 182.6 F g^-1^ at 1 A g^-1^ | 83.2% for 5000 cycles at 3 A g^-1^ | [31] |
| ZnCo_2_O_4_@NiCo_2_S_4_@PPy//AC | 0-1.7V | 3 M KOH | 109.9 F g^-1^ at 1 A g^-1^ | 80.7% for 3000 cycles at 5 A g^-1^ | [38] |


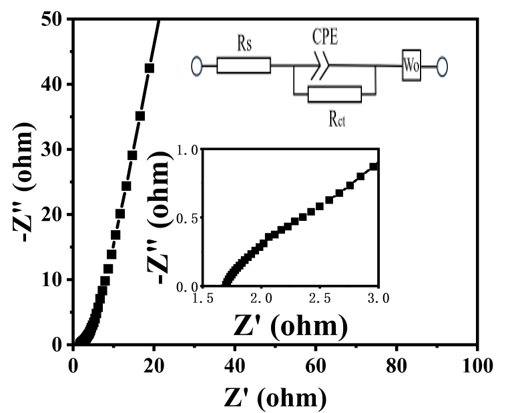


**Figure S30.** Nyquist curves of H-Fe_3_O_4_@C-15//H-NiCo_2_S_4_@C-40, the inset shows the details in high-frequency rangeb and electrical equivalent circuit.


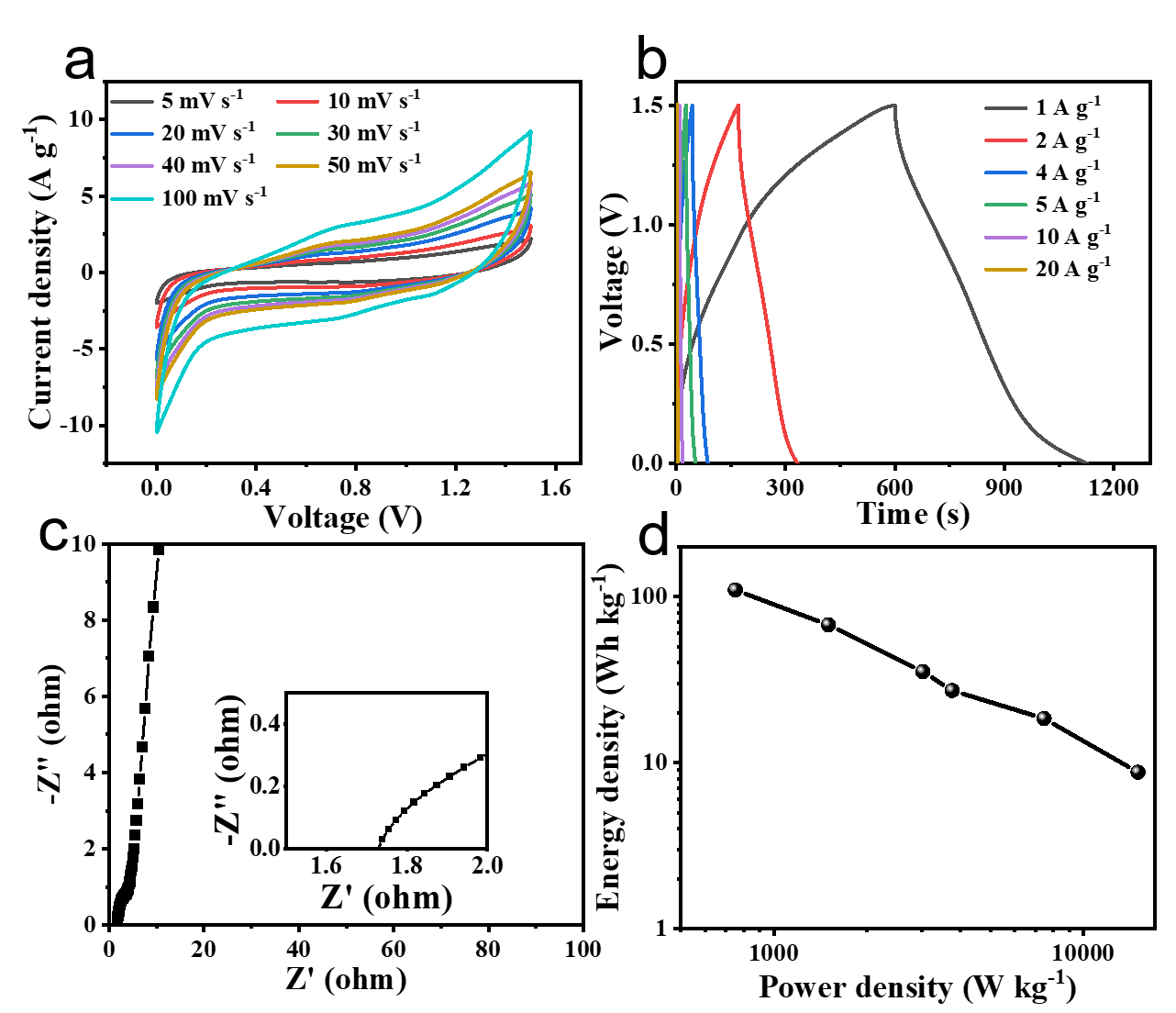


**Figure S31.** Electrochemical performances of the as-prepared asymmetric coin cells with 6.0 M KOH containing 0.03 M K_3_Fe(CN)_6_ electrolyte: (a,b) CV and GCD curves with various scan rates and current densities; (c) Nyquist plot; (d) Ragone plot of energy density versus power density.

**Table S10** Comparison of energy and power density with reported similar devices.

| **Materials** | **Energy density** | **Power density** | **Ref.** |
| --- | --- | --- | --- |
| **H-NiCo_2_S_4_@C-40//H-Fe_3_O_4_@C-15** | **105 Wh kg^-1^**  **46.9 Wh kg^-1^** | **749 W kg^-1^**  **14.9 kW kg^-1^** | **This work** |
| Fe_3_O_4_@C@PANi//Fe_3_O_4_@C@PANi | 32.7 Wh kg^-1^ | 500 W kg^-1^ | [17] |
| NiCo_2_S_4_@NiMoO_4_//AC | 21.4 Wh kg^-1^ | 58 W kg^-1^ | [45] |
| NiCo_2_S_4_@MnS//AC | 23.3 Wh kg^−1^ | 725 W kg^−1^ | [46] |
| Fe_3_O_4_@PPy@MnO_2_//AC | 71.3 Wh kg^−1^ | 750 W kg^−1^ | [21] |
| ZnCo_2_O_4_@NiCo_2_S_4_@PPy//AC | 44.15 Wh kg^−1^ | 850Wkg^−1^ | [47] |
| Fe_3_O_4_@MnO_2_//N-pC | 31.3 Wh kg^-1^ | 750 W kg^-1^ | [48] |
| NiCoO_2_@APPy//10h-Fe_3_O_4_/CNT-5 | 58.1 Wh kg^−1^ | 1007 Wkg^−1^ | [49] |
| Fe_3_O_4_@C//AC | 18.3 Wh kg^-1^ | 351 W kg^-1^ | [16] |
| NS-Fe_3_O_4_@N-PC-800//CNTs | 38.9 Wh kg^-1^ | 700.2 W kg^-1^ | [50] |
| NiCo_2_O_4_@NiMoO_4_//AC | 53.3 Wh kg^−1^ | 750Wkg^−1^ | [51] |


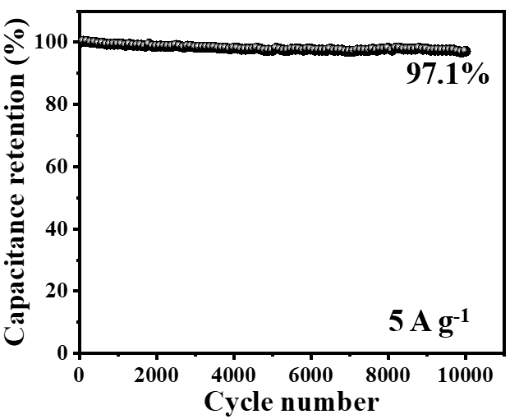


**Figure S32.** Cycling stability of the assembled ASC device at 5 A g⁻¹.


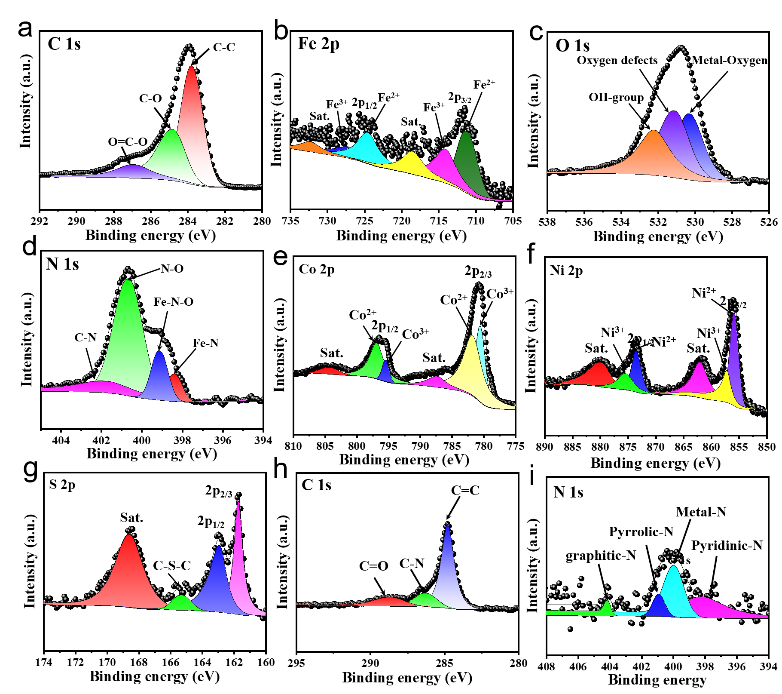


Figure S33. (a-d) The high-resolution XPS spectra of C 1s, Fe 2p, O 1s, N 1s for H-Fe_3_O_4_@C-15 after long-term cycling. (e-i) The high-resolution XPS spectra of Co 2p, Ni 2p, S 2p, C 1s N 1s for H-NiCo_2_S_4_@C-40 after long-term cycling.


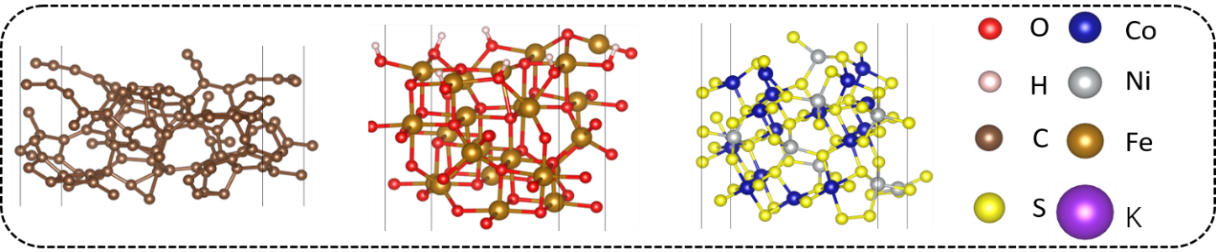


**Figure S34.** The structural models of AC, Fe_3_O_4_ and NiCo_2_S_4_.

**Table S11** List of K-mesh, lattice parameters, and convergence criterion used in DFT calculation.

|  | K-mesh | Lattice parameters | Convergence criterion on total energy in electronic step (eV) | Convergence criterion on force in ionic step (eV/Å) |
| --- | --- | --- | --- | --- |
| Fe_3_O_4_ bulk | 2×2×2 | *a*=*b=c*=8.53 Å,  *α*=*β*=*γ=*90° | 1×10^-5^ | 0.02 |
| NiCo_2_S_4_ bulk | 5×5×5 | *a*=*b=c*=9.31 Å,  *α*=*β*=*γ=*90° | 1×10^-5^ | 0.02 |
| AIMD of AC bulk structure |  |  | 1x10^-4^ | 0.03 |
| Optimization of AC bulk structure |  |  | 1×10^-4^ | 0.03 |
| AC slab surface with or without absorbate | 2×2×1 | *a*=8.54 Å,  *b=*13.45 Å,  *α*=*β*=90° | 1×10^-4^ | 0.03 |
| Fe_3_O_4_ (100) slab surface with or without absorbate | 2×2×1 | *a*=*b=*8.52 Å,  *α*=*β*= 90° | 1×10^-4^ | 0.03 |
| NiCo_2_S_4_ (311) slab surface with or without absorbate | 3×3×1 | *a*=11.40 Å,  *b=*6.58 Å,  *α*=*β*=90°,  γ=106.78° | 1×10^-5^ | 0.02 |

***References***

[1] L. Jing, K. Zhuo, L. Sun, N. Zhang, X. Su, Y. Chen, X. Hu, R. Feng, J. Wang, “The Mass-Balancing between Positive and Negative Electrodes for Optimizing Energy Density of Supercapacitors,” *Journal of the American Ceramic Society* 146 (2024): 14369, https://doi.org/ 10.1021/jacs.4c00486.

[2] J. F. b. G. Kresse, “Efficiency of ab-initio total energy calculations for metals and semiconductors using a plane-wave basis set,” *Computational Materials Science* 6 (1996): 15-50, https://doi.org/10.1016/0927-0256(96)00008-0.

[3] P. E. Blöchl, “Projector augmented-wave method,” *Physical Review B* 50 (1994): 17953, https://doi.org/10.1103/PhysRevB.50.17953.

[4] K. B. John P. Perdew, Matthias Ernzerhof, “Generalized Gradient Approximation Made Simple,” *Physical Review Letters* 77 (1996): 18, https://doi.org/10.1103/physrevlett.77.3865.

[5] S. Grimme, S. Ehrlich, L. Goerigk, “Effect of the damping function in dispersion corrected density functional theory,” *Journal of Computational Chemistry* 32 (2011): 1456, https://doi.org/ 10.1002/jcc.21759.

[6] R. Car, M. Parrinello, “Unified Approach for Molecular Dynamics and Density-Functional Theory,” *Physical Review Letters* 55 (1985): 2471, https://doi.org/ 10.1103/physrevlett.55.2471.

[7] J. Li, X. Yuan, C. Lin, Y. Yang, L. Xu, X. Du, J. Xie, J. Lin, J. Sun, “Achieving high pseudocapacitance of 2D titanium carbide (MXene) by cation intercalation and surface modification,” *Advanced Energy Materials* 7 (2017): 1602725, https://doi.org/ 10.1002/aenm.201602725.

[8] H. Zhang, X. Xin, H. Liu, H. Huang, N. Chen, Y. Xie, W. Deng, C. Guo, W. Yang, “Enhancing lithium adsorption and diffusion toward extraordinary lithium storage capability of freestanding Ti_3_C_2_T_x_ MXene,” *The Journal of Physical Chemistry C* 123 (2019): 2792, https://doi.org/10.1021/acs.jpcc.8b11255.

[9] S. Liu, Y. Yin, K. S. Hui, K. N. Hui, S. C. Lee, S. C. Jun, “High‐performance flexible quasi‐solid‐state supercapacitors realized by molybdenum dioxide@ nitrogen‐doped carbon and copper cobalt sulfide tubular nanostructures,” *Advanced Science* 5 (2018): 1800733, https://doi.org/10.1002/advs.201800733.

[10] X. Jiao, B. Li, J. Wang, Y. Fan, Y. Ma, Z. Yuan, C. Zhang, “Size-controllable synthesis of covalently interconnected few-shelled Fe_3_O_4_@onion-like carbons for high-performance asymmetric supercapacitors,” *Carbon* 203 (2023): 261, https://doi.org/10.1016/j.carbon.2022.11.053.

[11] R. Nasser, H. Zhou, H. Elhouichet, S. Melhi, Z. Li, J.-M. Song, “NiFe_2_O_4_@NiCo_2_O_4_ hollow algae-like microspheres enabled by Mott-Schottky for electrochemical energy storage” *Chemical Engineering Journal* 489 (2024): 151554, https://doi.org/10.1016/j.cej.2024.151554.

[12] L. Jin, X. Liu, Z. Wang, J. Luo, L. Zheng, M. Zhang, Y. Ao, “Fabrication of porous reduced graphene oxide encapsulated Cu(OH)_2_ core–shell structured carbon fiber-based electrodes for high-performance flexible supercapacitors,” *ACS Appl. Mater. Interfaces* 15 (2023): 58517, https://doi.org/10.1021/acsami.3c14872.

[13] K. A. Owusu, L. Qu, J. Li, Z. Wang, K. Zhao, C. Yang, K. M. Hercule, C. Lin, C. Shi, Q. Wei, L. Zhou, L. Mai, “Low-crystalline iron oxide hydroxide nanoparticle anode for high-performance supercapacitors,” *Nature Communications* 8 (2017): 14264, https://doi.org/10.1038/ncomms14264.

[14] G. Liu, B. Cai, Z. Hu, H. Gu, J. Zhou, R. Xu, Y. Liu, J. Xu, Y. Jiang, “Fe_2_O_3_@FeSe_2_ heterostructure as high-performance supercapacitor negative electrode material,” Journal of Energy Storage 88 (2024): 111544*,* https://doi.org/10.1016/j.est.2024.111544.

[15] S. Li, L. Zhang, L. Zhang, Y. Guo, X. Chen, R. Holze, T. Tang, “Preparation of Fe_3_O_4_@polypyrrole composite materials for asymmetric supercapacitor applications,” *New Journal of Chemistry* 45 (2021): 16011, https://doi.org/ 10.1039/d1nj02957f.

[16] H. Fan, R. Niu, J. Duan, W. Liu, W. Shen, “Fe_3_O_4_@ carbon nanosheets for all-solid-state supercapacitor electrodes,” *ACS Applied Materials & Interfaces* 8 (2016): 19475, https://doi.org/10.1021/acsami.6b05415.

[17] Z. Qiu, Y. Peng, D. He, Y. Wang, S. Chen, “Ternary Fe_3_O_4_@C@PANi nanocomposites as high-performance supercapacitor electrode materials,” *Journal of Materials Science* 53 (2018): 12322, https://doi.org/10.1007/s10853-018-2451-9.

[18] E. Payami, R. Teimuri-Mofrad, “A novel ternary Fe_3_O_4_@Fc-GO/PANI nanocomposite for outstanding supercapacitor performance,” *Electrochimica Acta* 383 (2021): 138296, https://doi.org/10.1016/j.electacta.2021.138296.

[19] S. Cui, F. Wang, K. Sun, X. Wang, Q. Hu, H. Peng, G. Ma, Z. Lei, “High-performance hybrid supercapacitors based on Ce-doped NiMoO_4_ nanosheets and Fe_3_O_4_@Bi_2_O_3_ nanoarrays,” *The Journal of Physical Chemistry C* 125 (2021): 18129, https://doi.org/10.1021/acs.jpcc.1c05387.

[20] S. Su, L. Lai, R. Wang, L. Zhang, Y. Cui, R. Li, N. Guo, W. Shi, X. Zhu, “One-step green and scalable dry synthesis of nitrogen-doped graphene-encapsulated Fe_3_O_4_ nanoparticles as high-performance supercapacitor electrode,” *Journal of Alloys and Compounds* 834 (2020): 154477, https://doi.org/ 10.1016/j.jallcom.2020.154477.

[21] L. Tong, C. Wu, J. Hou, X. Zhang, J. Yan, Z. Wang, Y. Wang, J. Mu, Z. Zhang, H. Che, “Fe_3_O_4_@PPy@MnO_2_ ternary core-shell nanospheres as electrodes for enhanced energy storage performance,” *Journal of Electroanalytical Chemistry* 922 (2022): 116725, https://doi.org/ 10.1016/j.jelechem.2022.116725.

[22] H. Wang, X. Xu, A. Neville, “In situ synthesis of nanostructured Fe_3_O_4_@TiO_2_ composite grown on activated carbon cloth as a binder-free electrode for high performance supercapacitors,” *RSC Advances* 11 (2021): 23541, https://doi.org/ 10.1039/d1ra04424a.

[23] P. Salarizadeh, M. B. Askari, K. Hooshyari, H. Saeidfirozeh, “Synergistic effect of MoS_2_ and Fe_3_O_4_ decorated reduced graphene oxide as a ternary hybrid for high-performance and stable asymmetric supercapacitors,” *Nanotechnology* 31 (2020): 435401, https://doi.org/ 10.1088/1361-6528/aba1bd.

[24] Q. Chen, W. Wei, J. Tang, J. Lin, S. Li, M. Zhu, “Dopamine-assisted preparation of Fe_3_O_4_@MnO_2_ yolk@ shell microspheres for improved pseudocapacitive performance,” *Electrochimica Acta* 317 (2019): 628, https://doi.org/ 10.1016/j.electacta.2019.06.011

[25] M. Guo, X. Liu, J. Du, Y. Cao, X. Li, Y. Zhang, “Synthesis, analysis and characterization of Mo-doped Fe_3_O_4_ nanoparticles decorated on rGO as an anode for high-performance supercapacitors,” *Journal of Materials Science: Materials in Electronics* 35 (2024): 1496, https://doi.org/ 10.1007/s10854-024-13264-2.

[26] Z. Li, Y. Zhang, W. Zhang, “Controlled synthesis of CNTs/MoS_2_/Fe_3_O_4_ for high-performance supercapacitors,” *Materials Research Express* 4 (2017): 055018, https://doi.org/ 10.1088/2053-1591/aa6c3f.

[27] X. Lv, G. Li, Z. Pang, D. Li, L. Lei, P. Lv, M. Mushtaq, Q. Wei, “Fabricate BC/Fe_3_O_4_@PPy 3D nanofiber film as flexible electrode for supercapacitor application” *Journal of Physics and Chemistry of Solids* 116 (2018): 153, https://doi.org/ https://doi.org/10.1016/j.jpcs.2018.01.012.

[28] X. Huang, L. Gou, “High performance asymmetric supercapacitor based on hierarchical flowerlike NiCo_2_S_4_@polyaniline,” *Applied Surface Science* 487 (2019): 68, https://doi.org/10.1016/j.apsusc.2019.05.005.

[29] L. Wan, C. He, D. Chen, J. Liu, Y. Zhang, C. Du, M. Xie, J. Chen, “In situ grown NiFeP@NiCo_2_S_4_ nanosheet arrays on carbon cloth for asymmetric supercapacitors,” *Chemical Engineering Journal* 399 (2020): 125778, https://doi.org/ 10.1016/j.cej.2020.125778.

[30] W. Zhu, T. Feng, M. Zhang, J. Tan, M. Wu, “Surface electronics regulation and enhanced electrochemical performance of nitrogen-doped carbon layer encapsulated NiCo_2_S_4_ nanosheets for supercapacitors,” *Vacuum* 220 (2024): 112729, https://doi.org/ 10.1016/j.vacuum.2023.112729.

[31] Y. Yu, J. Liu, L. Wang, L. Hou, “Preparation of MgCo_2_O_4_@NiCo_2_S_4_ core-shell nanocomposites for high-performance asymmetric supercapacitors,” *Electrochimica Acta* 439 (2023): 141664, https://doi.org/ 10.1016/j.electacta.2022.141664.

[32] Y. Sun, Y. Wang, C. Wang, J. Wang, Z. Wang, M. Zhang, H. Zong, J. Xu, J. Liu, “Construction of Co_9_S_8_@NiCo_2_S_4_ core–shell hetero-nanostructure with synergistic effect of abundant mesopores and multi-metallic elements for novel high-performance flexible hybrid supercapacitors,” *Chemical Engineering Journal* 469 (2023): 143812, https://doi.org/10.1016/j.cej.2023.143812.

[33] D. Wang, J. Wang, Y. Chu, S. Zha, Y. Chen, X. Du, N. Mitsuzaki, S. Jia, Z. Chen, “Crystalline NiCo_2_O_4_ and amorphous NiCo_2_S_4_ heterostructured electrode for high-performance asymmetric supercapacitors,” *Journal of Energy Storage* 96 (2024): 112614, https://doi.org/10.1016/j.est.2024.112614.

[34] Y. Li, Y. Zhao, S. Song, J. wang, “ynthesis of NiCo_2_S_4_@NiMoO_4_ Nanosheets with Excellent Electrochemical Performance for Supercapacitor” *Research Square* 10 (2021): 21203, https://doi.org/10.21203/rs.3.rs-641149/v1.

[35] Y. Ye, C. Yang, P. Chen, C. Ma, X. Chen, K. Guo, “Thorn-like nanostructured NiCo_2_S_4_ arrays anchoring graphite paper as self-supported electrodes for ultrahigh rate flexible supercapacitors,” *Electrochimica Acta* 399 (2021): 139420, https://doi.org/ 10.1016/j.electacta.2021.139420.

[36] J. Wang, Y. Xie, L. Wang, L. Wang, L. Yue, D. Jin, “Facile synthesis of morphology-controllable NiCo_2_S_4_ arrays on activated carbon textile as high-performance binder-free supercapacitor electrode,” *Materials Research Bulletin* 131 (2020): 110957, https://doi.org/ 10.1016/j.materresbull.2020.110957.

[37] J. Xie, Y. Yang, G. Li, H. Xia, P. Wang, P. Sun, X. Li, H. Cai, J. Xiong, “One-step sulfuration synthesis of hierarchical NiCo_2_S_4_@NiCo_2_S_4_ nanotube/nanosheet arrays on carbon cloth as advanced electrodes for high-performance flexible solid-state hybrid supercapacitors,” *RSC Advances* 9 (2019): 3041, https://doi.org/ 10.1039/c8ra10435b.

[38] J. Zhu, Y. Wang, X. Zhang, W. Cai, “MOF-derived ZnCo_2_O_4_@NiCo_2_S_4_@PPy core–shell nanosheets on Ni foam for high-performance supercapacitors,” *Nanotechnology* 32 (2021): 145404, https://doi.org/ 10.1088/1361-6528/abd20b.

[39] H. Li, X. Chen, E. Zalnezhad, K. N. Hui, K. S. Hui, M. J. Ko, “3D hierarchical transition-metal sulfides deposited on MXene as binder-free electrode for high-performance supercapacitors,” *Journal of Industrial and Engineering Chemistry* 82 (2020): 309, https://doi.org/10.1016/j.jiec.2019.10.028.

[40] Y. Li, X. Yan, W. Zhang, W. Zhou, Y. Zhu, M. Zhang, W. Zhu, X. Cheng, “Hierarchical micro-nano structure based NiCoAl-LDH nanosheets reinforced by NiCo_2_S_4_ on carbon cloth for asymmetric supercapacitor,” *Journal of Electroanalytical Chemistry* 905 (2022): 115982, https://doi.org/ 10.1016/j.jelechem.2021.115982.

[41] J. Lin, H. Liang, H. Jia, S. Chen, J. Guo, J. Qi, C. Qu, J. Cao, W. Fei, J. Feng, “In situ encapsulated Fe_3_O_4_ nanosheet arrays with graphene layers as an anode for high-performance asymmetric supercapacitors,” *Journal of Materials Chemistry A* 5 (2017): 24594, https://doi.org/10.1039/c7ta07628b.

[42] J. Zhao, Z. Li, X. Yuan, Z. Yang, M. Zhang, A. Meng, Q. Li, “A high‐energy density asymmetric supercapacitor based on Fe_2_O_3_ nanoneedle arrays and NiCo_2_O_4_/Ni(OH)_2_ hybrid nanosheet arrays grown on SiC nanowire networks as free‐standing advanced electrodes,” *Advanced Energy Materials* 8 (2018): 1702787, https://doi.org/ 10.1002/aenm.201702787.

[43] X. Huang, Y. Yang, J. Zhao, Y. Huang, X. Wang, “Formation of hierarchical core-shell hollow Co_3_S_4_@NiCo_2_S_4_ nanocages with enhanced performance for supercapacitor,” *Journal of Alloys and Compounds* 947 (2023): 169413, https://doi.org/ 10.1016/j.jallcom.2023.169413.

[44] X. Liang, H. He, X. Yang, W. Lü, L. Wang, X. Li, “In-situ growth of bimetallic sulfide NiCo_2_S_4_ nanowire on carbon cloth for asymmetric flexible supercapacitors,” *Journal of Energy Storage* 42 (2021): 103105, https://doi.org/10.1016/j.est.2021.103105.

[45] Y. Zhang, J. Xu, Y. Zheng, Y. Zhang, X. Hu, T. Xu, “NiCo_2_S_4_@ NiMoO_4_ core-shell heterostructure nanotube arrays grown on Ni foam as a binder-free electrode displayed high electrochemical performance with high capacity,” *Nanoscale Research Letters* 1 (2017): 12, https://doi.org/ 10.1186/s11671-017-2180-z.

[46] Z. Zhang, X. Huang, H. Li, H. Wang, Y. Zhao, T. Ma, “All-solid-state flexible asymmetric supercapacitors with high energy and power densities based on NiCo_2_S_4_@MnS and active carbon,” *Journal of Energy Chemistry* 26 (2017): 1260, https://doi.org/10.1016/j.jechem.2017.09.025.

[47] J. Zhu, Y. Wang, X. Zhang, W. Cai, “MOF-derived ZnCo_2_O_4_@NiCo_2_S_4_@PPy core–shell nanosheets on Ni foam for high-performance supercapacitors,” *Nanotechnology* 14 (2021): 32, https://doi.org/10.1088/1361-6528/abd20b.

[48] Q. Chen, W. Wei, J. Tang, J. Lin, S. Li, M. Zhu, “Dopamine-assisted preparation of Fe_3_O_4_@MnO_2_ yolk@ shell microspheres for improved pseudocapacitive performance,” *Electrochimica Acta* 317 (2019): 628, https://doi.org/ 10.1016/j.electacta.2019.06.011.

[49] S. H. Gong, J. Kuai, J. D. Wang, F. Liu, J. F. Wu, X. C. Wang, J. P. Cheng, “Fe_3_O_4_ nanoparticles anchored on carbon nanotubes as high-performance anodes for asymmetric supercapacitors,” *Nanotechnology* 34 (2023): 50, https://doi.org/10.1088/1361-6528/acf9af.

[50] M. Zhu, Q. Chen, J. Tang, W. Wei, S. Li, “Core@shell β-FeOOH@polypyrolle derived N, S-codoped Fe_3_O_4_@N-doped porous carbon nanococoons for high performance supercapacitors,” *Applied Surface Science* 480 (2019): 582, https://doi.org/ 10.1016/j.apsusc.2019.02.242.

[51] H. Zhang, C. Lu, H. Hou, Y. Ma, S. Yuan, “Tuning the electrochemical performance of NiCo_2_O_4_@NiMoO_4_ core-shell heterostructure by controlling the thickness of the NiMoO_4_ shell,” *Chemical Engineering Journal* 370 (2019): 400, https://doi.org/ 10.1016/j.cej.2019.03.168.
